# High endothelial venules (HEVs) in immunity, inflammation and cancer

**DOI:** 10.1007/s10456-021-09792-8

**Published:** 2021-05-06

**Authors:** Lucas Blanchard, Jean-Philippe Girard

**Affiliations:** Institut de Pharmacologie et de Biologie Structurale, IPBS, Université de Toulouse, CNRS, UPS, Toulouse, France

**Keywords:** High endothelial venules (HEVs), Lymphocyte trafficking, Chronic inflammatory diseases, Cancer immunology, Tumor blood vessels, Tertiary lymphoid structures

## Abstract

High endothelial venules (HEVs) are specialized blood vessels mediating lymphocyte trafficking to lymph nodes (LNs) and other secondary lymphoid organs. By supporting high levels of lymphocyte extravasation from the blood, HEVs play an essential role in lymphocyte recirculation and immune surveillance for foreign invaders (bacterial and viral infections) and alterations in the body’s own cells (neoantigens in cancer). The HEV network expands during inflammation in immune-stimulated LNs and is profoundly remodeled in metastatic and tumor-draining LNs. HEV-like blood vessels expressing high levels of the HEV-specific sulfated MECA-79 antigens are induced in non-lymphoid tissues at sites of chronic inflammation in many human inflammatory and allergic diseases, including rheumatoid arthritis, Crohn’s disease, allergic rhinitis and asthma. Such vessels are believed to contribute to the amplification and maintenance of chronic inflammation. MECA-79^+^ tumor-associated HEVs (TA-HEVs) are frequently found in human tumors in CD3^+^ T cell-rich areas or CD20^+^ B-cell rich tertiary lymphoid structures (TLSs). TA-HEVs have been proposed to play important roles in lymphocyte entry into tumors, a process essential for successful antitumor immunity and lymphocyte-mediated cancer immunotherapy with immune checkpoint inhibitors, vaccines or adoptive T cell therapy. In this review, we highlight the phenotype and function of HEVs in homeostatic, inflamed and tumor-draining lymph nodes, and those of HEV-like blood vessels in chronic inflammatory diseases. Furthermore, we discuss the role and regulation of TA-HEVs in human cancer and mouse tumor models.

## Introduction

**Fig. 1:**
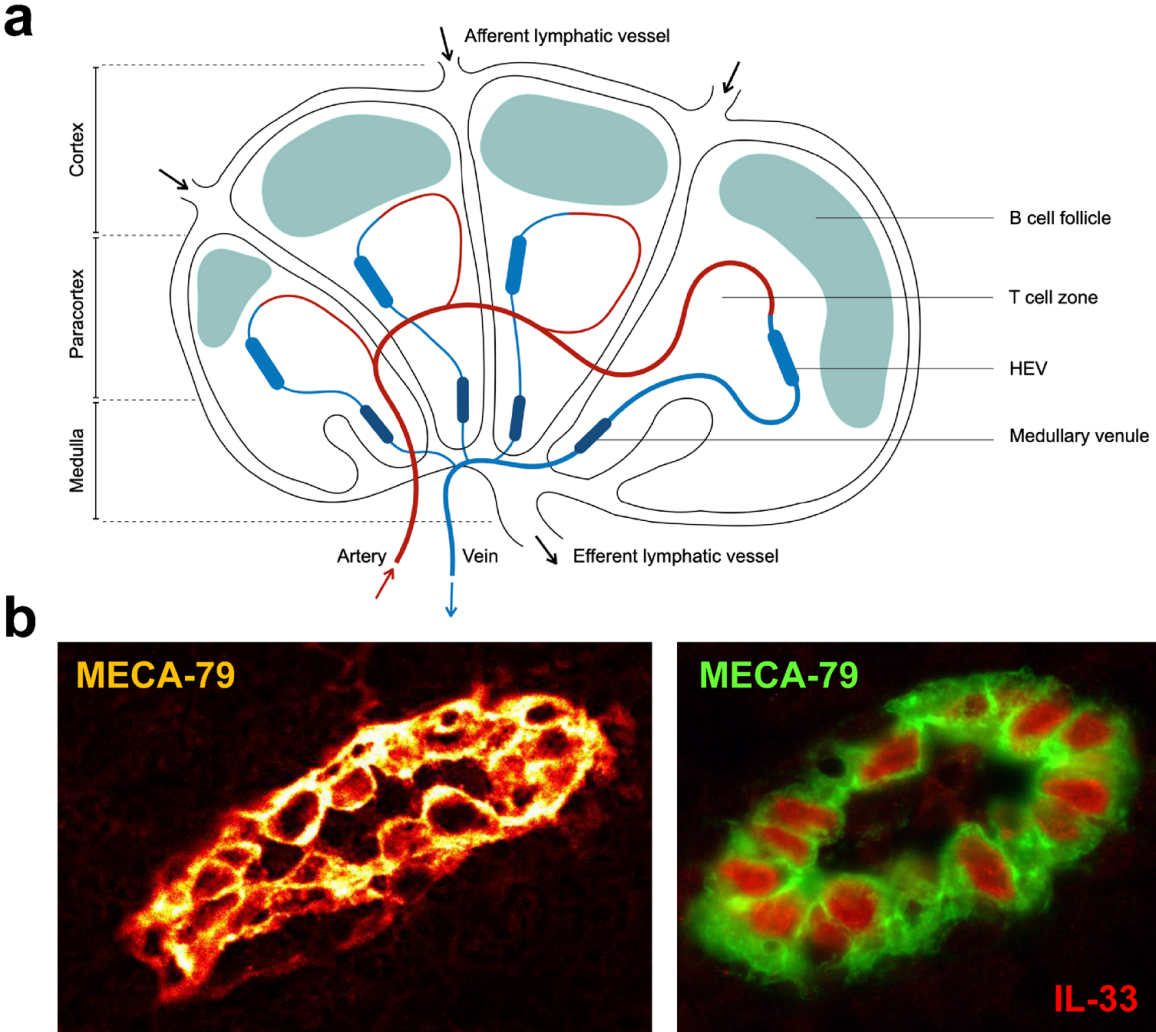
HEVs in secondary lymphoid organs. **a** Lymph nodes are encapsulated lymphoid organs subdivided into three regions: the cortex, the paracortex and the medulla. Blood enters the LN through a main feeding artery that branches into arterioles and capillaries in the medulla and the paracortex, respectively. Then, blood flows from the capillary beds into the post-capillary HEVs that are located in the T cell zone of the LN. Finally, blood flows through medullary venules and leave the LN via a collecting vein. Immune cells enter the LN through HEVs or afferent lymphatic vessels and exit via the efferent lymphatic vessel in the medulla. **b** HEVs in human tonsils. MECA-79 staining reveals the “plump” cuboidal morphology of HEV endothelial cells (HECs) (Left). MECA-79^+^ HECs express high levels of the nuclear cytokine IL-33 [7] (Right). The gene encoding IL-33 was originally discovered as a gene highly expressed in MECA-79^+^ HECs isolated from human tonsils, and IL-33 was thus initially designated as “nuclear factor from high endothelial venules” (NF-HEV) [8, 9]

Endothelial cells play critical roles in physiology and physiopathology, and are involved in many important diseases, including cardiovascular diseases, chronic inflammatory diseases and cancer. Although all vascular endothelial cells share certain common functions, considerable structural and functional heterogeneity exists along the length of the vascular tree and in the microvascular beds of various organs. One of the most striking examples of organ-specific endothelial cell differentiation occurs at the level of high endothelial venules (HEVs), specialized post-capillary venules found in lymph nodes (LNs) and other secondary lymphoid organs (Fig. 1) which mediate high levels of lymphocyte extravasation from the blood [1–6].

The most obvious characteristic of HEV endothelial cells (HECs) revealed by light-microscopic examination is their morphology. HECs have a plump, almost cuboidal appearance very different from the flat appearance of endothelial cells that line other vessels. This cuboidal appearance provides the basis for the name of high endothelium. Thome first noted the plump morphology of HECs in LNs in 1898 [10]. Thome wrote that, “at first notice, one is more inclined to think of the duct of a gland rather than that of a blood vessel”. A few months later, in 1899, von Schumacher confirmed the observations of Thome and noted the presence of numerous lymphocytes within HEV walls [11]. However, the direction and physiological significance of lymphocyte migration through HEVs remained unappreciated during many decades. In two landmark studies published in 1964, Gowans et al. showed that radioactively labeled lymphocytes injected intravenously migrated rapidly into rodent LNs by crossing HEV walls [12, 13]. Gowans concluded that HEVs are the site of a large-scale migration of lymphocytes from the blood into secondary lymphoid organs. Indeed, HEV-mediated recruitment of lymphocytes is a very efficient process. It is estimated that as many as 5 × 10^6^ lymphocytes migrate through the HEVs of the human body every second [2].

HEVs are present in all secondary lymphoid organs with the exception of spleen, including hundreds of LNs dispersed in the body, tonsils, adenoids, Peyer’s patches in the small intestine, appendix, and small aggregates of lymphoid tissue associated with the mucosal surfaces of the respiratory, gastrointestinal and urogenital tract. HECs range from 7 to 10 μm in width and 5–7 μm in height and are much less regular in outline than the term cuboidal would suggest. They exhibit great deformability and irregularity of shape [14]. The increased height of HECs might permit them to close about lymphocytes migrating through intercellular spaces, thus allowing lymphocytes to cross the endothelium like “ships in canal locks” with minimal vascular leakage [15]. Although the most striking feature of HECs is their unusual height, ultrastructural analysis revealed additional features generally not observed in endothelial cells from other vessels. At the ultrastructural level, HECs exhibit the characteristics of metabolically active secretory-type cells, with a prominent Golgi complex, abundant mitochondria closely associated with rough endoplasmic reticulum, many ribosomes frequently found in polyribosome clusters, and a large rounded nucleus with one or two nucleoli [14, 16]. The Golgi is particularly developed in areas where lymphocyte crossing is intense and often oriented towards the transmigrating lymphocytes [17]. HEV ligands for L-selectin, the major lymphocyte homing receptor, pass through the Golgi apparatus during their biosynthesis and become reactive to L-selectin in large Trans-Golgi-Network-associated vesicles [18]. After crossing the Golgi, these L-selectin ligands “en route” to the HEV lumen are present in cytoplasmic vesicles, likely to represent secretory vesicles. Another remarkable feature of HECs revealed by ultrastructural studies is the thick carbohydrate-rich glycocalyx that coats their luminal surface and represents the true interface with circulating lymphocytes [2, 16, 19]. Direct measurements showed that the thickness of the glycocalyx varied from 490 ± 12 in capillaries to 1280 ± 108 Å in HEVs [16]. This feature of the HEV glycocalyx is noteworthy in view of the evidence that sulfated carbohydrates and glycoproteins serve as essential recognition determinants for lymphocyte L-selectin [6]. In addition, the HEV glycocalyx may also facilitate the retention (immobilization) of secreted molecules on the luminal surface of HEVs [2, 14]. Indeed, immobilization of chemokines by heparan sulfate is important for HEV-mediated lymphocyte entry into LNs [20, 21].

Evidence accumulated over the past 40 years indicates that blood vessels with HEV features develop in non-lymphoid tissues in many human chronic inflammatory diseases [2, 6, 22]. During the 1980s, Freemont and Ziff visualized the presence of HEV-like blood vessels in areas of lymphocyte aggregation in the inflamed synovium of patients suffering from rheumatoid arthritis (RA) [23, 24]. Such vessels distinguished by the plump morphology of their endothelial cells, and the presence of numerous lymphocytes within their walls, were not present in normal synovium. In their pioneering studies, Freemont and Ziff observed a strong correlation between the “plumpness” of endothelial cells lining HEV-like blood vessels and the number and percentage of perivascular lymphocytes [23, 24]. Jalkanen, Butcher, and Freemont demonstrated the capacity of HEV-like blood vessels to support lymphocyte adhesion in frozen sections of the inflamed synovium in vitro and to incorporate large amounts of radioactive sulfate, a unique metabolic property shared with lymph node HEVs [25, 26]. Together, these observations suggested that lymphocytes emigrated through HEV-like blood vessels to enter the inflamed synovium during RA. Freemont extended his observations to many other human chronic inflammatory diseases [27]. He showed that HEV-like blood vessels with cuboidal endothelium, that mediated sulfate uptake and lymphocyte adhesion in vitro, were present in areas of lymphocyte infiltration (> 150 lymphocytes/mm^2^) in many tissues and disease states [22]. Freemont made several important observations: HEV-like blood vessels developed in sites that did not contain such vessels under normal conditions; lymphocyte infiltration always preceded the development of these vessels; plump endothelial cells did not show mitotic activity. Based on these observations, he concluded that HEV-like blood vessels develop from existing vessels following lymphocyte infiltration, and once developed, participate in a positive feedback loop increasing lymphocyte extravasation into the diseased tissues, thus contributing to the amplification and maintenance of chronic inflammation [22].

Ten years ago, we reported that blood vessels with HEV characteristics are frequently found in the stroma of many human solid tumors including melanomas, breast, ovarian, colon and lung carcinomas [28, 29]. These findings extended initial observations made by Freemont in the 1980′s [30]. In both breast tumors (*n* = 273) and primary melanomas (*n* = 225), the density of tumor-associated HEVs (TA-HEVs) was highly correlated with the density of CD3^+^ T cells (including CD8^+^ cytotoxic T cells) and CD20^+^ B cells, indicating that TA-HEVs may function as major portals of entry for lymphocytes into human solid tumors [28, 29]. Interestingly, a high density of TA-HEVs in the tumor microenvironment significantly correlated with longer survival of breast cancer patients [28]. Blood vessels and tumor angiogenesis promote tumor growth and are generally associated with unfavorable clinical outcome. Therefore, these studies introduced the concept that “the phenotype of tumor blood vessels is important and that some subsets of tumor blood vessels (i.e. TA-HEVs) can contribute to tumor suppression rather than tumor growth” [28]. Studies in other human tumor types and murine tumor models confirmed these initial observations in primary breast cancer and melanoma (see below). Together, the findings suggested that TA-HEVs represent attractive targets for cancer diagnosis and treatment, and that novel therapeutic strategies based on the modulation of TA-HEVs could have a major impact on antitumor immunity and clinical outcome of cancer patients.

There are comprehensive reviews about the role of HEVs in LNs and other secondary lymphoid organs, to which the reader is referred [1–6]. In our previous article, we reviewed the phenotype and function of HEVs in LNs at steady state [1]. In the present review, we highlight the role and regulation of HEVs in homeostatic, inflamed and tumor-draining LNs, and those of HEV-like blood vessels in chronically inflamed tissues, and TA-HEVs in human and mouse tumors.

## High endothelial venules and lymphocyte trafficking in lymph nodes

### MECA-79^+^ HEVs in homeostatic lymph nodes (LNs)

In mammals, HEVs are present not only in LNs and other secondary lymphoid organs [1–6] but also in unconventional lymphoid tissues such as nasopharyngeal-associated lymphoid tissue (NALT) [31–33], tear duct-associated lymphoid tissue (TALT) [34], intestinal isolated lymphoid follicles (ILF) [35], mediastinal fat-associated lymphoid clusters (FALC) [36], and omental milky spots [37, 38]. The precise phenotype and function of HEVs in these various lymphoid tissues go beyond the scope of this review. Butcher et al. previously highlighted differences between HEVs in peripheral LNs, mucosal LNs and Peyer’s patches [3, 39, 40]. Since HEV biology has been mostly studied in peripheral LNs [1], we will focus our discussion in the following paragraphs on peripheral lymph node HEVs. Today, we have a good understanding of the molecular mechanisms regulating lymphocyte extravasation through HEVs, thanks to the major contributions of the groups of Rosen, Butcher, Von Andrian, Miyasaka, Fukuda, Cyster, Lowe and many others [1–6].

The first interaction between naive lymphocytes and HEVs is initiated by lymphocyte L-selectin (also known as CD62L) that recognizes a family of sulfated mucin-like glycoproteins known as HEV sialomucins [1, 6]. Although not specific to HECs, these HEV sialomucins, which include CD34, podocalyxin, endomucin, nepmucin and glycosylation-dependent cell adhesion molecule 1 (GlyCAM-1, only present in rodents; pseudogene in humans), become effective L-selectin ligands when they are post-translationally modified by enzymes highly expressed in HECs. For instance, CD34 that is broadly expressed on endothelial cells along the vascular tree, as well as on hematopoietic progenitors, functions as an L-selectin counter-receptor only when appropriately decorated by HEC-specific sulfated, fucosylated and sialylated oligosaccharides [41, 42]. The critical carbohydrate determinant for L-selectin recognition, 6-sulfo sialyl Lewis^X^ (sialic acidα2-3Galβ1-4(Fucα1-3(sulfo-6)GlcNAcβ1-R), is abundantly produced in HEVs and is present on both *N*-glycans and extended core 1 and 2 *O*-glycans decorating HEV sialomucins [6, 43–49]. The expression of high levels of the L-selectin-binding HEV-specific glycoforms of HEV sialomucins is undoubtedly one of the most important features of the HEV endothelium. Indeed, monoclonal antibodies (mAbs) that define the best HEV markers currently available are directed against HEV-specific oligosaccharides decorating HEV sialomucins [43–46, 50]. For instance, the HEV-specific mAb “Mouse Endothelial Cell Antigen-79” (MECA-79), generated by Butcher et al. in 1988 [50], specifically recognizes 6-sulfo sialyl Lewis^X^ structures on extended core 1 *O*-glycans [45]. The MECA-79 epitope is often designated peripheral lymph node addressin (PNAd) [50, 51]. However, it is important to mention that MECA-79 reacts not only with peripheral LN HEVs, but also with mucosal LN HEVs, Peyer’s patches HEVs and HEV-like blood vessels in non-lymphoid tissues. Therefore, although the term PNAd is widely used to designate MECA-79 reactive antigens in both lymphoid and non-lymphoid organs, MECA-79^+^ antigens and MECA-79^+^ blood vessels may be more appropriate designations than PNAd outside of peripheral LNs. MECA-79 is a fantastic tool for HEV studies. It is a very robust mAb for immunohistochemistry and immunofluorescence studies, which reacts specifically with HEVs in both humans and mice (no cross-reaction with other blood vessels in the body), and in both lymphoid and non-lymphoid organs [50, 51]. Importantly, this is a function-blocking mAb that inhibits interactions of lymphocytes with HEVs in vitro and in vivo [50, 52].

Crucial insights about the HEV phenotype came from genome-wide transcriptomic analyses of HECs from mouse peripheral LNs [40, 53, 54] that extended pioneering studies of isolated human and mouse MECA-79^+^ HECs by differential expression and subtractive hybridization strategies [55–58]. MECA-79^+^ HECs displayed a unique transcriptional program clearly distinct from that of all other endothelial cell subsets in the LN [40, 53, 54]. Single-cell RNA sequencing (scRNA-seq) revealed that genes encoding HEV sialomucin GlyCAM-1 (*Glycam1*), CC-chemokine ligand 21 (CCL21; *Ccl21a*), and critical HEV enzymes (sulfotransferases: *Chst4, Chst2*; glycosyltransferases: *Fut7, Gcnt1, B3gnt3, St3gal6*) [45, 47–49, 59, 60], were among the top genes differentially and highly expressed in HECs [53]. In contrast, genes encoding other HEV sialomucins (*Cd34, Emcn,* …), were not differentially expressed in HECs. These transcriptomic analyses confirmed that the unique capacity of HECs to capture large numbers of lymphocytes is based on the coordinated expression of the different enzymes involved in the decoration of HEV sialomucins with high affinity 6-sulfo sialyl Lewis^X^ L-selectin ligands [40, 53]. Transcriptional analysis also confirmed high expression in HECs of several genes implicated in HEV function (*Enpp2, Spns2, Sphk1*) that were previously or subsequently identified through in vivo studies in mice [61–65]. Another striking feature of HECs revealed by scRNA-seq is their cellular heterogeneity in homeostatic LNs [53]. Indeed, the two most abundant HEV genes in mouse peripheral LNs, *Glycam1* and *Ccl21a* exhibit differential expression in HECs. In a subset of HEVs, some MECA-79^+^ HECs expressed high levels of *Glycam1* (or *Ccl21a*) mRNAs, although adjacent cells expressed none [53]. We also observed spatial heterogeneity of HEVs. MECA-79^+^ HECs in the LN paracortex had higher expression levels of GlyCAM-1 protein than MECA-79^+^ HECs located close to the medulla. The functional consequences of this HEC heterogeneity are currently unknown, but it reveals the highly plastic nature of the HEV phenotype. The spatial localization of HECs within the LN microenvironment might dictate accessibility to factors regulating HEV gene and protein expression and thereby contribute to the remarkable heterogeneity of HECs at steady state.

### HEV-mediated entry of lymphocytes in lymph nodes

**Fig. 2:**
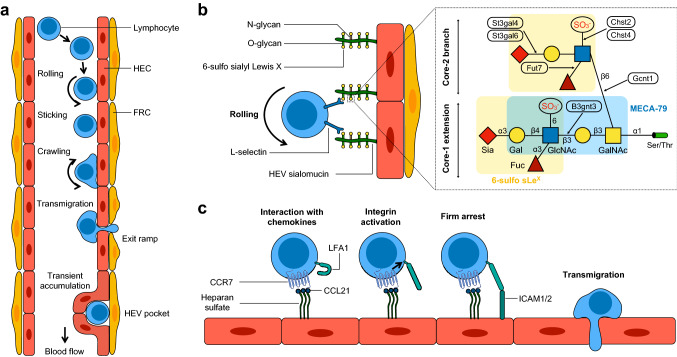
HEV-mediated recruitment of lymphocytes in peripheral lymph nodes. **a** Naive T and B cells circulating in the blood tether and roll on HEV walls. Subsequently, rolling lymphocytes interact with chemokines immobilized on the HEV luminal surface. Chemokine receptor-dependent signaling induces activation of lymphocyte integrins that mediate firm binding (sticking) to their counter-receptors on HEV endothelium. Then, lymphocytes crawl on the HEV surface for a few minutes before transmigrating across the HEV endothelium via “exit ramps”. Some lymphocytes also accumulate transiently in “HEV pockets”. **b** Naive lymphocytes roll on HEV endothelium through the binding of L-selectin to 6-sulfo sialyl Lewis X motifs decorating both *O*-glycans and *N*-glycans on HEV sialomucins (Left). Representation of a bi-antennary O-linked glycan on a HEV sialomucin (Right). Both extended core-1 and core-2 branch structures can display the 6-sulfo sialyl Lewis X motif (highlighted in yellow). The 6-sulfo sialyl Lewis X motif is a tetra-saccharide composed of *N*-acetylglucosamine (GlcNAc), galactose (Gal), sialic acid (Sia) and fucose (Fuc), linked through *N*-acetylgalactosamine (GalNAc) to a serine (Ser) or threonine (Thr) residue of the core HEV sialomucin protein. α and β linkages of the saccharide units are shown. The epitope of MECA-79 (highlighted in blue) is a component of the core-1 extension. The C-6 sulfation (red SO3-) of *N*-acetylglucosamine, that is referred to as “6-sulfo”, is required for both L-selectin and MECA-79 recognition. Black rectangles indicate genes encoding enzymes involved in the synthesis of the 6-sulfo sialyl Lewis X motif. **c** Naive lymphocytes rolling on HEV walls interact with chemokines that are presented by heparan sulfate such as CCL21. Signaling through CCR7 induces conformational changes in the lymphocyte integrin LFA1, which mediate binding to ICAM1 and ICAM2 on the HEV endothelium, leading to firm arrest (sticking) of the lymphocytes. Following a rapid step of crawling, lymphocytes eventually transmigrate through HEVs to enter the lymphoid tissue

In homeostatic LNs, HEVs almost exclusively recruit naive and central memory lymphocytes [1]. Migration of naive T and B cells through HEVs, which has been precisely described thanks to the intravital microscopy technique set up by von Andrian in 1996 [52], occurs via a multistep adhesion cascade composed of rolling, firm arrest (sticking) and transmigration (Fig. 2a) [1, 52, 66]. Lymphocytes circulating in the blood first tether and roll on HEV walls through the binding of L-selectin to 6-sulfo sialyl Le^X^ motifs decorating HEV sialomucins (Fig. 2b). This initial tethering interaction significantly reduces the velocity of lymphocytes, allowing them to interact with chemokines immobilized and presented on the luminal surface of HEVs by heparan sulfate [20, 21]. Homeostatically expressed chemokines CCL21, CCL19, CXCL13 and to a lesser extent CXCL12, are pivotal factors during lymphocyte migration across HEVs because they mediate the activation of integrins essential for lymphocyte arrest in HEVs [67–71]. Indeed, while L-selectin is constitutively active, integrins require prior activation to recognize their ligands and subsequently mediate firm adhesion (sticking) to endothelial cells. Naive T cells express CC-chemokine receptor 7 (CCR7) and CXC-chemokine receptor 4 (CXCR4), the receptors for CCL21, CCL19 and CXCL12, whereas B cells express CXC-chemokine receptor 5 (CXCR5), the receptor for CXCL13, in addition to CXCR4 and CCR7 [72, 73]. Endothelium-presented chemokines are either produced by HEVs (such as CCL21 in mice but not in humans) or produced by neighboring stromal cells (fibroblastic reticular cells (FRCs) and follicular dendritic cells (FDCs)) and then transcytosed through HEVs [73–75].

The integrin lymphocyte function-associated antigen 1 (LFA1), which binds to intercellular adhesion molecule 1 and 2 (ICAM1 and ICAM2) expressed on endothelial cells, is the major integrin for T and B cell arrest in peripheral LN HEVs. In mesenteric LNs and other gut-associated lymphoid tissue (GALT), including Peyer’s patches, the integrin α4β7, a major ligand of mucosal addressin cell adhesion molecule 1 (MAdCAM-1), is also critical for lymphocyte recruitment through HEVs [6, 76]. The combination of shear forces of the blood flow and G protein-coupled chemokine receptor signaling induces conformational changes in LFA1 molecules, leading to firm adhesion of lymphocytes to ICAM1 and ICAM2 expressed on HECs (Fig. 2c) [68, 70, 77]. Following stable arrest, lymphocytes can be observed crawling along the luminal surface of HEVs, looking for appropriate transmigration sites [78]. When they finally find an exit site, lymphocytes rapidly cross the endothelial barrier via paracellular (between adjacent endothelial cells) or transcellular (through the cytoplasm of endothelial cells) migration even if the paracellular migration route seems to be largely favored [79–83]. Remarkably, lymphocytes tend to follow each other through discrete hot spots that are called “exit ramps” when transmigrating through HEVs [78]. However, crawling lymphocytes can also transiently accumulate in endothelial structures called HEV pockets before entering the LN parenchyma [83, 84]. These “waiting areas” could be instrumental in homeostatic LNs to maintain a constant steady-state cellularity while supporting extensive lymphocyte trafficking. In addition to the mechanisms described above, other adhesion molecules implicated in lymphocyte trans-endothelial migration exist. For additional information, readers are referred to “state-of-the-art” reviews on leukocyte transmigration [85, 86].

### Mechanisms regulating the phenotype and function of HEVs in lymph nodes

Pioneering studies performed more than 30 years ago revealed that LN afferent lymphatic vessel ligation results in HEV dedifferentiation [87]. This process, which involves HEV morphology “flattening”, downregulation of MECA-79 antigens and reduced ability to support lymphocyte adhesion, is fully revertible following interruption of ligation [88–90]. Interestingly, subsequent studies demonstrated that lymph-borne molecules such as chemokines can reach HEVs through a stromal conduit system composed of FRCs, revealing a special connection between HEVs and the lymph coming from drained tissues [75, 91, 92]. Together with phenotypic analyses showing that freshly purified human HECs rapidly lose the specialized HEV phenotype when cultured ex vivo [93], these results indicated that HEVs exhibit a remarkable plasticity and are highly dependent on the lymphoid microenvironment and lymph-derived cells and/or factors.

Eventually, we discovered that CD11c^+^ dendritic cells (DCs) are critical for maintenance of HEV phenotype and function in homeostatic LNs [94]. Indeed, in vivo depletion of CD11c^+^ DCs induces a reversion to an immature HEV phenotype characterized by reduced expression of MECA-79 antigens, downregulation of HEV-specific genes (*Chst4, Fut7, Glycam1*) and upregulation of the mucosal addressin MAdCAM-1, a marker of immature HEVs in neonatal peripheral LNs [95]. The functional consequence of this altered HEV phenotype is a profound defect in lymphocyte recruitment to LNs that culminates in LN hypocellularity. Additional studies confirmed the pivotal role of DCs in HEV-mediated lymphocyte homing to LNs [96, 97]. Interestingly, it has also been shown that DCs contribute to HEV growth in a vascular endothelial growth factor (VEGF)-dependent fashion, which confers additional regulating properties to DCs [96, 98].

The lymphotoxin-β receptor (LTβR) and downstream non-canonical nuclear factor kB (NF-kB) signaling pathway are essential for HEV maintenance and lymphocyte homing to adult LNs [99–102]. Endothelial cell-specific deletion of LTβR and treatment with LTβR-immunoglobulin (Ig) soluble decoy receptor indicate that continuous triggering of LTβR on HECs is critical for the expression of several genes related to HEV biology (*Glycam1, Fut7, Chst4, Gcnt1*), demonstrating that many HEV-specific genes are LTβR-dependent genes [53, 99, 102, 103]. scRNA-seq analyses after treatment with LTβR-Ig revealed that *Chst4* requires lower levels of LTβR-dependent signals for expression than the other HEV genes (*Glycam1, Fut7, Gcnt1*) [53]. LTβR stimulation results in activation of both canonical and non-canonical NF-kB signaling pathway whereas tumor necrosis factor receptor 1 (TNFR1) engagement mediates canonical NF-kB signaling only [104]. Neither the phenotype of HEVs nor the expression of HEV-specific genes are affected in TNFR1-deficient mice and mice treated with TNFR-Ig [99, 101]. On the contrary, LN HEVs from mice deficient in components of the non-canonical NF-kB signaling pathway have reduced expression of MECA-79 antigens, GlyCAM-1 and GlcNAc6ST-2 (*Chst4*), showing that LTβR ability to induce the non-canonical NF-kB signaling pathway is essential for the regulation of HEVs [100, 101].

In fact, we demonstrated that CD11c^+^ DC are a major source of LTβR ligands, lymphotoxin α (LTα), lymphotoxin β (LTβ) and LIGHT, and that DC-derived lymphotoxin is critical for HEV-mediated lymphocyte recruitment to homeostatic LNs [94]. Because intranodal DCs are positioned close to HEVs both at steady-state and during inflammation [105–107], we proposed a model in which DCs regulate HEV phenotype and function through direct stimulation of LTβR [1]. Future studies will be required to identify the precise DC subsets involved in the process although LN-resident conventional DC 1 and 2 (cDC1 and cDC2) appear as obvious candidates because of their frequent association with HEVs [107].

### HEVs in inflamed lymph nodes

**Fig. 3:**
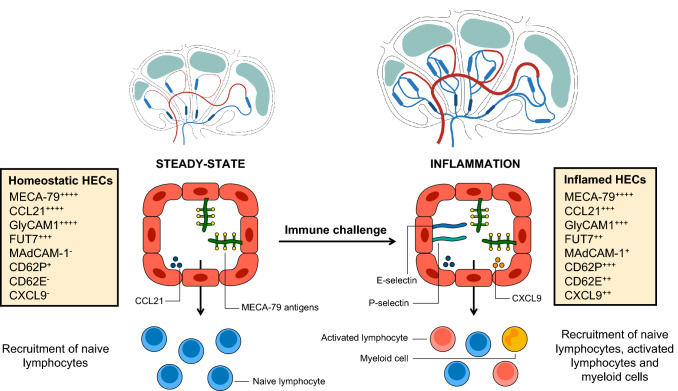
HEVs in inflamed lymph nodes. During an immune challenge, the HEV network expands, contributing to the increase in size and cellularity of the reactive LN (Top). HECs in reactive LNs are phenotypically different from HECs in homeostatic LNs at steady state (Bottom). scRNA-seq analyses revealed the precise phenotypes of homeostatic and inflamed LN HECs [53]. Several genes encoding inflammatory proteins are upregulated (P- and E-selectins, CXCL9), resulting in the recruitment of novel immune cells such as activated lymphocytes and myeloid cells. Importantly, the recruitment of naïve lymphocytes in inflamed LNs is still efficient despite downregulation of mature HEV genes, probably because levels of MECA-79 antigens and chemokine CCL21 remain very high

LNs can be regarded as immune hubs strategically positioned in the organism to provide regional immune surveillance [108]. These highly specialized organs orchestrate the initiation and the maintenance of adaptive immune responses during infection and cancer. Following immune challenge, the LN draining inflamed tissues is the site of an important stromal remodeling enabling its increase in size and cellularity [109, 110]. Within the inflamed LN, the number but also the phenotype of HEVs are modified to support the ongoing immune response (Fig. 3) [53, 98, 103, 111, 112].

Soon after the initial inflammatory stimulus, the LN blood vasculature undergoes substantial enlargement and remodeling which includes expansion of the primary feed arterioles and HEV network, a process that is thought to increase influx of lymphocytes and therefore the efficiency of screening for rare antigen-specific lymphocytes [112–114]. Mechanistically, it has been shown in models of multicolor fate mapping that LN blood vascular growth relies on the clonal proliferation of some HECs that act as local progenitors to create both capillaries and HEV neo-vessels [112]. More recently, scRNA-seq studies of mouse LN endothelial cells identified a population of progenitor-like activated capillary endothelial cells, defined as capillary resident precursors (CRPs), that are actively mobilized for LN angiogenesis after immunization [54]. Among LN BECs, CRPs selectively express Apln and can be observed by staining for the human estrogen receptor (ER), which serves as a surrogate for Apln expression. At steady state, ER^+^ endothelial cells are present in capillaries whereas HECs do not express the receptor. However, Apln-reporter mice revealed that many HECs and capillary endothelial cells are positive for the reporter three weeks after an immune challenge, showing that Alpln-expressing CRPs can also contribute to inflammation-induced HEV neogenesis, in addition to HECs themselves [112].

DC mobilization increases during the initial phase of LN swelling [115]. DC accumulation in the inflamed LN could be the initial trigger for blood vasculature enlargement as DCs have been shown to control proliferation of endothelial cells (including HECs) in LNs [98, 116], although B and T cells may participate too [117–119]. In both instances, LTβR ligands and VEGF-A are the critical mediators of LN vasculature remodeling. At later stages, afferent lymphatic function is transiently diminished, likely causing dilution of DCs in the LN microenvironment [103]. Concurrently to this afferent lymph flow shutdown, HECs acquire an inflamed endothelial cell phenotype (Fig. 3) that is marked by temporary downregulation of mature HEV genes (*Glycam1, Fut7, Gcnt1*), maintenance of strong MECA-79 expression, and upregulation of inflammatory proteins (P-selectin, E-selectin and CXCL9) and immature HEV marker MAdCAM-1 [53, 103, 120–123]. As a consequence of this phenotypic switch, novel immune cell populations such as neutrophils and activated effector/effector memory T cells are recruited through inflamed HEVs [121, 122, 124–127]. Importantly, the ability of inflamed HEVs to mediate L-selectin-dependent naive lymphocyte recruitment is not compromised despite downregulation of mature HEV genes [53, 120]. Interestingly, recent work from the Butcher’s lab revealed that non-HEV medullary post-capillary venules could also be involved in myeloid cell homing to inflamed LNs via an L-selectin-independent mechanism, unveiling the existence of local venular specializations for the recruitment of specific immune cell populations during acute inflammation of the LN [54].

### HEVs in tumor-draining lymph nodes

The tumor-draining lymph node (tdLN), which is the first regional lymph node draining established tumors, is considered as the major activation site of tumor-specific lymphocytes [128]. The TdLN is not only important for the initiation of T-cell-dependent antitumor responses, but also for response to various cancer treatments, including radiotherapy and immune checkpoint blockade [129, 130]. However, as a sentinel LN, the tdLN is also a privileged site for cancer cell metastasis, revealing its dual role in cancer [131, 132].

Because they mediate naive lymphocyte entry to tdLNs, HEVs indirectly participate to the priming of naive lymphocytes specific for cancer antigens and are consequently crucial components of T-cell-dependent antitumor responses. In fact, HEV-mediated homing of naive lymphocytes to tdLNs is even targeted by the primary tumor which reduces expression of CCL21 on HEVs, thereby reducing lymphocyte adhesion to the endothelium [133]. This process is a striking illustration of the capacity of the primary tumor to drive HEV reprogramming in the tdLN [132]. Indeed, several reports in mouse models and human patients indicate that tdLN HEVs exhibit extensive phenotypical and morphological changes during tumor progression, including vessel dilatation, thinning of HEC morphology and discontinuous expression of MECA-79 antigens [134–138]. TdLN HEV remodeling occurs before the apparition of nodal metastases, suggesting that it is part of a pre-metastatic niche establishment program induced by the primary tumor [134–136]. Nevertheless, the density of abnormal HEVs is significantly higher in patients with established metastases in their LNs, showing that HEV identity and function might by highly compromised in metastatic LNs [135]. In some instances, the level of HEV remodeling in the tdLN correlated with disease progression and clinical outcome [135, 136]. For instance, abnormal HEVs with red blood cells observed in their lumen, which is a feature of HEVs with altered vascular integrity [139], have been associated with a worse prognosis in squamous cell carcinoma [135].

The participation of the tdLN in the dissemination of cancer cells to distant organs is a widely accepted hypothesis [131]. Efferent lymphatics and subsequent passage through thoracic duct is the major dissemination route for cancer cells, but the ability of abnormal HEVs to provide extra-lymphatic route of dissemination has also been questioned [140]. Two recent studies based on intralymphatic injection of high numbers of cancer cells in afferent lymphatics concluded that LN HEVs could constitute an effective exit route for cancer cell dissemination in the blood circulation [141, 142]. However, intralymphatic injection of non-physiologic numbers of cancer cells might not accurately mimic the metastatic processes observed in human patients, thus challenging the clinical relevance of these results. Moreover, whether incriminated HEVs are *bona fide* HEVs or profoundly abnormal venules that have lost their HEV function remains unclear. Metastasis is more likely to occur through de-differentiated HEVs that are no longer functional for lymphocyte recruitment.

## HEV-like blood vessels in chronic inflammatory diseases

### MECA-79^+^ HEV-like blood vessels in chronically inflamed tissues

**Fig. 4:**
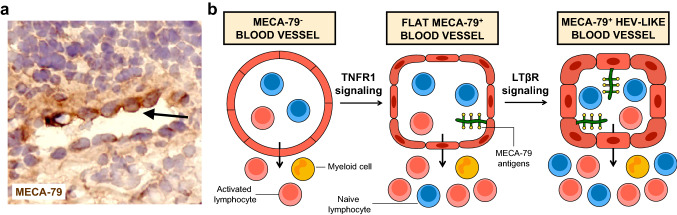
HEV-like blood vessels in chronic inflammation. **a** A MECA-79^+^ HEV-like blood vessel in the inflamed synovium from a patient suffering from RA. Endothelial cells exhibit a “plump” cuboidal morphology. Staining with MECA-79 is more intense on the side of the vessel in contact with the immune infiltrate (arrow). **b** During acute inflammation, MECA-79^−^ blood vessels are able to recruit activated lymphocytes and myeloid cells (Left). Prolonged inflammatory signals (such as LTα3) trigger TNFR1 signaling that induces expression of MECA-79 antigens on post-capillary venules lined by flat endothelial cells, during the initial stages of chronic inflammation (Middle). Maintenance of chronic inflammation and subsequent activation of LTβR signaling induce additional maturation and acquisition of a fully mature HEV-like phenotype that is associated with increased luminal expression of MECA-79 antigens, cuboidal morphology and enhanced recruitment of naive lymphocytes (Right)

Inflammation is an evolutionary conserved process characterized by the activation of immune and non-immune cells to protect the host from foreign invaders during tissue injury, infection and cancer [143]. Acute inflammation is a temporally restricted protective response that is rapidly resolved to limit excessive tissue damage. In contrast, chronic inflammation is a persistent and non-resolving response causing tissue destruction and loss of function with progressive clinical symptoms. Immune cell-induced reprogramming of stromal cells is an important feature of chronic inflammation and is thought to exacerbate inappropriate immune responses [144]. HEV-like blood vessels phenotypically similar to lymphoid tissue HEVs appear in many human inflammatory diseases affecting different anatomic sites (Table 1), including chronic inflammatory diseases such as RA (Fig. 4a) and inflammatory bowel diseases (Crohn’s disease, ulcerative colitis), and allergic diseases such as asthma and allergic rhinitis [2, 6, 145–148]. Thus, development of HEV-like blood vessels is not disease- or organ-specific and might be a universal property of chronically inflamed tissues.

**Table 1:** MECA-79^+^ HEV-like blood vessels in human inflammation

Condition	Target organ	Associated features
*Allergic diseases*		
Bronchial asthma [149, 150]	Lung	Co-expression of sLex epitope HECA-452
Allergic rhinitis [151]	Nasal mucosa	
Allergic contact dermatitis [51]	Skin	
*Chronic inflammatory diseases*		
Rheumatoid arthritis [51, 152–156]	Synovium	Disappearance after anti-TNFα treatment; Expression of GlcNAc6ST-2 (CHST4); Co-expression of sLex epitope HECA-452; Perivascular stromal cells producing CCL21; Presence of TLSs in high-grade inflammatory lesions
Inflammatory bowel diseases (Crohn’s disease, ulcerative colitis) [152, 155, 157–162]	Gut	Disappearance during remission in ulcerative colitis; Associated with T_N_ and T_CM_ infiltration Preferentially associated with T cells, particularly CD4^+^ T cells; Co-expression of sLex epitope HECA-452; Perivascular stromal cells producing CCL21; Presence of TLSs
Autoimmune thyroiditis (Hashimoto’s disease, Graves’ disease) [51, 157]	Thyroid	Co-expression of sLex epitope HECA-452
Arthritis [163]	Synovium	
Spondyloarthritis [164]	Skeleton	Disappearance after anti-IL-17A treatment (Secukinimab)
Inflammatory skin diseases (psoriasis, lichen planus, cutaneous lymphoid hyperplasia, cutaneous lupus erythematosus) [51, 157, 165, 166]	Skin	Lymphoid infiltrates but not organized in TLSs; Co-expression of sLex epitope HECA-452
Conjunctival inflammation [167]	Conjunctiva	Not reduced after hydrocortisone treatment
Chronic rhinosinusitis [168, 169]	Nasal and paranasal mucosa	Associated with severity of inflammation
Sjögren’s syndrome [155, 170, 171]	Salivary glands	Perivascular stromal cells producing CCL21; Presence of TLSs
Lichen planus [165]	Oral mucosa	Preferentially associated with T cells, particularly CD4+ T cells
Type I autoimmune pancreatitis [172]	Pancreas	
Inflammatory myopathies [173]	Muscle	Presence of TLSs
Bronchiectasis [174]	Lung	
Idiopathic pulmonary arterial hypertension [175]	Lung	Presence of TLSs
Glomerulonephritis [176]	Kidney	
*Infection*		
Chronic *Helicobacter pylori* gastritis [235–237]	Stomach	Associated with progression of inflammation; Disappearance after eradication of *H. pylori*
*Organ transplant rejection*		
Acute heart allograft rejection [177, 178]	Heart	Associated with severity of graft rejection; Co-expression of sLex epitope HECA-452; Presence of TLSs
Acute kidney allograft rejection [179, 180]	Kidney	Co-expression of sLex epitope HECA-452; Presence of TLSs
Obliterative bronchiolitis after lung transplantation [181]	Lung	
*Hyperplasia and benign neoplasms*		
Warthin’s tumor [182]	Salivary gland	Preferentially associated with T cells
Benign prostatic hyperplasia [183]	Prostate	Preferentially associated with T cells, particularly CD4^+^ T cells; Associated with severity of inflammation and lower urinary tract symptoms
Cutaneous pseudolymphomas [184]	Skin	
*Pregnancy*		
Pregnant uterus [185]	Decidua	Reduced density of MECA-79^+^ HEV-like blood vessels is associated with idiopathic recurrent pregnancy losses

*HECA-452* mAb recognizing non-sulfated sLex, *T*_*CM*_ central memory T cells, *T*_*N*_ naive T cells, *TLSs* B cell-rich tertiary lymphoid structures

The HEV-specific mAb MECA-79 recognizes HEVs from lymphoid tissues, but also HEV-like blood vessels from extra-lymphoid tissues in both mice and humans, making it a very useful tool for the identification of ectopic HEV-like blood vessels. Indeed, systematic surveys involving large numbers of independent samples revealed that HEV-like blood vessels are recognized by MECA-79 in various human chronic inflammatory diseases affecting many different organs [51, 157]. MECA-79^+^ HEV-like blood vessels express the post-capillary venule marker Duffy antigen receptor for chemokines (DARC) [186], similar to HEVs in lymphoid organs [56], suggesting that they likely arise from inflammation-induced reprogramming of pre-existing post-capillary venules [152]. HEV-like blood vessels also express the mucosal addressin MAdCAM-1 in GALT during inflammatory bowel diseases [187]. In many diseases, the intensity of MECA-79 staining correlated with the extent of mononuclear cell infiltration in inflamed lesions, which suggests that the level of expression of MECA-79 antigens might be a good indicator of the functional competence of HEV-like blood vessels. Interestingly, some flat-walled blood vessels are positive for MECA-79, indicating that MECA-79^+^ blood vessels encompass a wide range of venules with distinct degree of maturation regarding the HEV phenotype. Histological examinations of HEV-like blood vessels in human chronically inflamed tissues precludes definitive conclusions on their functionality and their ability to mediate lymphocyte recruitment. In contrast, mouse models of chronic inflammation, which recapitulate several features of human diseases including the development of HEV-like blood vessels, allow for in vivo functional investigations. A comprehensive list of mouse inflammatory conditions in which MECA-79^+^ HEV-like blood vessels develop is included in Table 2. AKR mice develop hyperplastic thymus containing MECA-79^+^ HEV-like blood vessels in close association with T and B cells, before the onset of T cell lymphoma [188]. Short-term in vivo homing assays showed that MECA-79^+^ HEV-like blood vessels are involved in lymphocyte trafficking to the hyperplastic thymus. Indeed, injection of blocking amounts of MECA-79 or anti-L-selectin mAb MEL-14 abolished the recruitment of adoptively transferred lymphocytes, revealing the functional significance of MECA-79 expression on HEV-like blood vessels [188]. Similar findings were obtained in the inflamed lacrimal glands of NOD mice, a model for autoimmune-mediated insulin-dependent diabetes mellitus (IDDM) in which ectopic lymphoid infiltrates containing MECA-79^+^ HEV-like blood vessels are observed in several tissues [189]. After seven months of life, NOD mice also develop bronchus-associated lymphoid tissue (BALT) in the lung. Interestingly, treatment with MECA-79 and MEL-14 antibodies blocked the homing of adoptively transferred lymphocytes from blood into inflamed bronchopulmonary tissues [190]. These results obtained in three distinct inflamed tissues demonstrate first, that MECA-79^+^ HEV-like blood vessels are functional, and second, that the L-selectin-MECA-79 antigens axis is involved in lymphocyte trafficking to various chronically inflamed tissues. In contrast to MECA-79 antigens, MAdCAM-1 is not involved in the recruitment of adoptively transferred lymphocytes to the inflamed lacrimal glands and BALT of NOD mice [189, 190].

**Table 2:** MECA-79^+^ HEV-like blood vessels in mouse inflamed tissues

Condition	Target organ	Associated features
*Models of chronic inflammation*		
Diabetes (NOD mice) [191–193]	Pancreas	Co-expression of MAdCAM-1; Expression of the HEV-restricted sulfotransferase GlcNAc6ST-2 (Chst4)
Autoimmune sialoadenitis (NOD mice) [191, 192, 194]	Salivary gland	Expression of the HEV-restricted sulfotransferase GlcNAc6ST-2 (Chst4); Reduced after LTβR-Ig treatment; Presence of TLSs
Autoimmune dacryoadenitis (NOD mice) [189, 191, 195]	Lacrimal gland	Expression of the HEV-restricted sulfotransferase GlcNAc6ST-2 (Chst4); MECA-79 and anti-CD62L block migration of adoptively transferred lymphocytes to inflamed lacrimal glands; Reduced after LTβR-Ig treatment; Presence of TLSs
BALT (NOD mice) [190]	Lung	MECA-79 and anti-CD62L block migration of adoptively transferred B and T lymphocytes to BALT; Presence of TLSs
Thymic hyperplasia (AKR mice) [188, 191, 196]	Thymus	Co-expression of MAdCAM-1; Expression of the HEV-restricted sulfotransferase GlcNAc6ST-2 (Chst4) and fucosyltransferase Fuc-T7 (Fut7); Associated with binding of L-selectin-IgM chimera; MECA-79 and anti-CD62L block migration of adoptively transferred lymphocytes to hyperplastic thymus
Neonatal thymectomy-induced autoimmune gastritis [197]	Gastric mucosa	Presence of TLSs
Diabetes (H8 mice derived-DC injection in RIP-LCMV-GP mice) [198]	Pancreas	Presence of TLSs
Collagen-induced arthritis [199]	Synovial tissue	Expression of the HEV-restricted sulfotransferase GlcNAc6ST-2 (Chst4)
Pristane-induced peritoneum inflammation [200]	Peritoneum	Presence of TLSs
Atherosclerosis (apoE^−/−^ mice) [201]	Aorta	Associated with migration of adoptively transferred lymphocytes; Reduced after LTβR-Ig treatment; Presence of TLSs
LPS-induced iBALT [202]	Lung	Present in Rorc^−/−^ and Id2^−/−^ mice; Absent in LTα^−/−^ and DKO mice, and after LTβR-Ig treatment; Presence of TLSs
Sialoadenitis (submandibular gland administration of AdV5) [203]	Salivary gland	Presence of TLSs
Bleomycin-induced lung fibrosis [36]	Lung	Presence of TLSs
Lupus nephritis (NZB/W lupus-prone mice) [204]	Kidney	Presence of TLSs
Skin inflammation (intradermal injection of newborn lymph node-derived cells) [205]	Skin	Absent with LTα^−/−^ mice-derived cells; Presence of TLSs
Skin inflammation (subcutaneous injection of lymph node-derived stromal cell lines) [206]	Skin	Presence of TLSs
*Infection*		
*Probionibacterium acnes*-induced granulomatous liver disease [207]	Liver	Presence of TLSs
*Helicobacter*-induced chronic hepatitis [208]	Liver	Co-expression of MAdCAM-1; Expression of CCL21; Presence of TLSs
*Helicobacter pylori*-induced gastritis [209]	Gastric mucosa	Presence of TLSs
Influenza-induced iBALT [210, 211]	Lung	Present in CXCL13^−/−^ mice; Reduced in plt/plt mice; Absent in LTα^−/−^ mice; Presence of TLS
*Genetically modified mice*		
Hyperplastic pancreatic islets (RIP1-Tag5 mice) [212]	Pancreas	
Inflammed pancreatic islets (RIP-CCL19 mice) [213]	Pancreas	Presence of TLSs
Inflammed pancreatic islets (RIP-CCL21, RIP-CCL21a and RIP-CCL21b) [213–215]	Pancreas	Present in Ikaros^−/−^ mice but absent in Rag1^−/−^ mice, and reduced after LTβR-Ig treatment; Presence of TLSs
Inflammed pancreatic islets (RIP-CXCL13) [191, 216]	Pancreas	Present in TNFR1^−/−^ mice but reduced in µM^−/−^ and LTα^−/−^ mice, and after LTβR-Ig treatment; Expression of the HEV-restricted sulfotransferase GlcNAc6ST-2 (Chst4); Presence of TLSs
Inflammed pancreatic islets (RIP-LT mice) [217, 218]	Pancreas	Absent in Rag2^−/−^ and p55^−/−^ (TNFR1) mice; Reduced infiltration of naive lymphocytes in LTβ^−/−^ mice; Presence of TLSs
Inflammed pancreatic islets (RIP-LTαβ mice) [219]	Pancreas	MECA-79^+^ HEV-like blood vessels with luminal expression of MECA-79 antigens and expression of the HEV-restricted sulfotransferase GlcNAc6ST-2 (Chst4); Present in LTβ^−/−^ mice; Presence of TLSs
Autoimmune pancreatitis (Tg(Ela1-LTα,β) mice) [220, 221]	Pancreas	Presence of TLSs; Reduced after LTβR-Ig treatment
Inflammed thyroid (TG-CCL21 mice) [222–224]	Thyroid	Present in Id2^−/−^; Absent in Rag1^−/−^ mice and phenotypic rescue with adoptive transfer of CD4^+^ T cells; MECA-79^+^ HEV-like blood vessels with only abluminal expression of PNAd, flat morphology and no expression of the HEV-restricted sulfotransferase GlcNAc6ST-2 (Chst4) in LTα^−/−^ mice; Absent after LTβR-Ig treatment; Presence of TLSs
*Organ transplant rejection*		
Cardiac allografts [225, 226]	Heart	Reduced after LTβR-Ig treatment; Presence of TLSs; Present in TLSs and outside TLSs in lymphocyte-rich areas

*AdV5* replication-defective adenovirus 5, *BALT* bronchus-associated lymphoid tissue, *CCL19* CC-chemokine ligand 19, *DKO* mice lacking the chemokines CXCL13, CCL19 and CCL21a, *H8 mice* transgenic mice constitutively expressing the LCMV immunodominant epitope GP33, *iBALT* inducible BALT, *LCMV-GP* lymphocytic choriomeningitis virus glycoprotein, *LPS* lipopolysaccharide, *LT* lymphotoxin α, *LTαβ* lymphotoxin α and β, *NOD* non-obese diabetic, *NZB/W* New Zealand black × New Zealand white F1 mice, *plt/plt* mice lacking CCL19 and CCL21a, *RIP* rat insulin promoter, *TG* thyroglobulin, *TLSs* B cell-rich tertiary lymphoid structures, *µM* B-cell-deficient mice

HEV-like blood vessels of chronically inflamed tissues can be observed close to diffuse non-organized lymphoid infiltrates, but they are also frequently associated with highly organized lymphoid clusters defined as tertiary lymphoid structures (TLSs) (Table 2) [6, 147, 227]. TLSs, also known as tertiary lymphoid organs (TLOs) [228], demonstrate several features of lymphoid organs, including compartmentalization of B and T cells in discrete zones, presence of dendritic cells and formation of HEV-like blood vessels [147]. By mediating lymphocyte entry into TLSs, HEV-like blood vessels may be critical for their maintenance and their function. The development of TLSs, referred to as lymphoid neogenesis [147, 148, 227], is observed in various chronic inflammatory diseases and is generally associated with deleterious outcomes in patients [148, 228, 229]. Indeed, several lines of evidence indicate that TLSs not only recapitulate the cellular and structural organization of lymphoid tissues, but can also support immune functions. In particular, TLSs can contain active germinal centers that foster B cell responses in situ [230–232], suggesting that TLSs might be regarded as B cell-oriented structures, at least regarding functional aspects.

### Mechanisms regulating the development of HEV-like blood vessels in chronic inflammation

HEV-like blood vessels are nearly always present when pronounced lymphocyte infiltration is present over the course of chronic inflammation, suggesting an important role of lymphocytes in the development of these specialized blood vessels. Interestingly, a growing body of evidence indicate that several mechanisms occurring during the development of physiological HEVs in lymphoid tissues are involved in the development of ectopic HEV-like blood vessels, with an especially strong participation of cytokines and chemokines.

As mentioned in the introduction, initial morphometric studies made by Ziff with electron microscopy revealed that HEV-like blood vessel “plumpness”, which can be regarded as a surrogate of HEV maturity, is associated with the number of perivascular lymphocytes [24]. Another feature highlighting the close relationship between HEV-like blood vessels and lymphocytes is the increased intensity of MECA-79 staining of HEV-like endothelial cells localized close to the lymphocytic infiltrates (Fig. 4a). These observations suggested that HEV-like blood vessel development could be the consequence of lymphocyte infiltration in chronically inflamed tissues. Subsequent studies further documented the intimate relationship between HEV-like blood vessels and lymphocytes [149, 157]. A striking finding is the influence of the nature of the immune infiltrate on the presence or absence of HEV-like blood vessels in diseases occurring in the same organs. For instance, MECA-79^+^ HEV-like blood vessels are induced in the skin and lungs during diseases associated with lymphocyte infiltration, such as psoriasis and bronchial asthma, but they are absent during diseases characterized by neutrophil infiltration, like vasculitis in the skin or adult respiratory distress syndrome in the lung [149, 157]. These observations suggest that HEV-like blood vessel induction is a hallmark of lymphocyte infiltration in chronically inflamed tissues, and that lymphocytes might be regulating their development and maintenance.

Mouse models of chronic inflammation have been instrumental for the identification of the mechanisms regulating HEV-like blood vessel development (Table 2). A major contribution of lymphoid tissue-associated cytokines and chemokines is strongly supported by results obtained in transgenic mice in which MECA-79^+^ HEV-like blood vessels are induced in pancreatic islets or thyroid in response to ectopic expression of TNF/lymphotoxin cytokines, CCL21 or CXCL13 [213, 214, 216, 217, 219]. LTα and LTβ have a central role in HEV development during LN organogenesis [233], and a similar scenario may apply for the de novo induction of HEV-like blood vessels. However, the phenotype of ectopic HEVs vary depending on the nature of the stimulus. When LTα is overexpressed in pancreatic islets, MECA-79^+^ HEV-like blood vessels exhibit mostly abluminal expression of MECA-79 antigens due to the absence of the sulfotransferase GlcNAc6ST-2 (*Chst4*) responsible for MECA-79 luminal expression [191, 234], and their development is dependent on TNFR1 signaling [218, 219]. On the other hand, co-expression of LTα and LTβ and consequent LTβR signaling in the exocrine pancreas is associated with the development of MECA-79^+^ HEV-like blood vessels expressing GlcNAc6ST-2 and high levels of luminal MECA-79 antigens [219]. These landmark studies of Ruddle et al. indicate that TNFR1 signaling is sufficient to initiate the formation of MECA-79^+^ HEV-like blood vessels but that LTβR signaling is required to generate vessels with increased HEV maturity and that might have an improved ability to capture L-selectin-expressing lymphocytes (Fig. 4b). Chemokines may induce HEV neogenesis through the recruitment of lymphocytes expressing LTβR ligands. Indeed, MECA-79^+^ HEV-like blood vessels induction in pancreatic islets following CCL21 or CXCL13 ectopic expression is abolished or reduced in lymphocyte-deficient mice [214, 216]. Moreover, crossing these transgenic mice with LTα^−/−^ mice or treating them with LTβR-Ig significantly reduces HEV-like blood vessel development [213, 216]. In fact, it was demonstrated that CCL21 and CXCL13 upregulate LTβR ligands on CD4^+^ T cells and B cells, respectively [213], showing that chemokines cooperate with TNF/lymphotoxin cytokines for the induction of HEV-like blood vessels in chronic inflammation. Interestingly, MECA-79^+^ HEV-like blood vessels induced in the thyroid after ectopic expression of CCL21 are lost in lymphocyte-deficient mice but can be rescued following adoptive transfer of CD4^+^ T cells [222, 223]. These later results confirmed that lymphocytes are critical regulators of HEV-like blood vessels, but also suggested that CD4^+^ T cells, that are preferentially associated with HEV-like blood vessels in several human chronically inflamed tissues [158, 159, 165, 183], are major inducers of HEV-like blood vessels in chronic inflammation.

### Therapeutic targeting of HEV-like blood vessels in chronic inflammation

Accumulating evidence indicates that HEV-like blood vessels induced at sites of chronic inflammation contribute to lymphocyte trafficking in the diseased tissue in a manner similar to lymphocyte homing in LNs. These specialized blood vessels sustain chronic inflammation and subsequent pathology. Therefore, their therapeutic targeting may offer a novel way of influencing the progression of chronic inflammation and could have broad applications because MECA-79^+^ HEV-like blood vessels appear in many distinct human inflammatory diseases (Table 1).

The presence of MECA-79^+^ HEV-like blood vessels correlates with the progression of inflammation and disease severity in several human inflammatory pathologies. In *Helicobacter pylori* chronic gastritis, MECA-79^+^ HEV-like blood vessels are likely to contribute to the formation of mucosa-associated lymphoid tissue (MALT) of the gastric mucosa that fosters local tissue inflammation and increases the risk of extranodal marginal zone lymphoma of MALT type (MALT lymphoma) [235–237]. By examining more than 140 human specimens, Fukuda and coworkers demonstrated that MECA-79^+^ HEV-like blood vessels positively correlated with the progression of inflammation in the gastric mucosa [235]. Furthermore, they showed that eradication of *H. pylori* by treatment with antibiotics and a proton pump inhibitor is associated with the disappearance of HEV-like blood vessels and minimal lymphocyte infiltration, suggesting that local post-capillary venules reacquire a normal phenotype after treatment and are no longer able to sustain extensive lymphocyte recruitment. Therapeutic agents also have an impact on HEV-like blood vessels in other diseases. For instance, in RA and psoriatic arthritis, reduced inflammation in the synovium after TNFα blockade with different biological agents (adalimumab, infliximab, etanercept) was associated with reduced numbers of MECA-79^+^ HEV-like blood vessels [153, 238]. Inflammatory bowel diseases (IBDs) such as ulcerative colitis provide another example of inflammatory pathologies in which HEV-like blood vessels are involved and modulated during disease progression [152, 157–161]. Analysis of colonic mucosa biopsies representing both active and remission phases of ulcerative colitis revealed that MECA-79^+^ HEV-like blood vessels are preferentially induced in the active phase of the disease [158, 161]. Finally, similar clinical correlations were observed in other human inflammatory disorders including begnin prostatic hyperplasia, chronic maxillary rhinosinusitis and acute heart allograft rejection [168, 177, 183], indicating that HEV-like blood vessels are tightly associated with persistent inflammation and active disease in humans.

Interfering with the development and/or maintenance of HEV-like blood vessels or with HEV-associated molecules controlling lymphocyte recruitment is likely to provide therapeutic benefits in many human inflammatory diseases (Table 1). In fact, several reports mention disease amelioration following therapeutic manipulation of HEV-like blood vessels in preclinical models. In a sheep model of human asthma associated with development of MECA-79^+^ HEV-like blood vessels in the lung, Rosen et al. showed that intravenous administration of MECA-79 antibody prevents airway hyper-responsiveness induced by allergen challenge and inhibits the accumulation of leukocytes in bronchoalveolar lavage fluid [150]. These results provided the first evidence that direct targeting of MECA-79^+^ HEV-like blood vessels can have therapeutic efficacy [150]. Similar findings were obtained in the same model using an anti-L-selectin mAb instead of MECA-79 [239]. Interestingly, blockade of L-selectin function has been associated with reduced leukocyte recruitment in various inflammatory conditions [240] and can inhibit insulitis and subsequent development of diabetes in NOD mice [241]. Another relevant approach for HEV-like blood vessel inhibition is the targeting of the HEV master regulator LTβR. In NOD mice, inhibition of MECA-79^+^ HEV-like blood vessels after treatment with LTβR-Ig is associated with improved function of salivary and lacrimal glands [194, 195], suggesting that LTβR inhibition may ameliorate disease in human Sjögren’s syndrome. Interestingly, LTβR-Ig reduces development of MECA-79^+^ HEV-like blood vessels in several murine tissue sites, including inflamed pancreatic islets, heart transplant allografts, and inflamed aorta during atherosclerosis in *apoE*^*−/−*^ mice (Table 2) [201, 216, 225]. However, a human LTβR-Ig fusion protein (Baminercept) failed to produce significant clinical efficacy in RA and Sjögren’s syndrome [242, 243]. The unique targeting of lymphocyte recruitment to inflamed tissues may thus not be sufficient to yield therapeutic benefits. The simultaneous targeting of distinct steps of the lymphocyte-dependent response with combination of different treatments will likely provide maximal therapeutic benefits in humans.

## Tumor-associated HEVs (TA-HEVs) in cancer immunology and immunotherapy

### MECA-79^+^ TA-HEVs in tumors

Although detrimental in chronic inflammatory diseases, the development of HEV-like blood vessels can be advantageous in other instances where increased lymphocyte recruitment is beneficial. The immune response against cancer is critically dependent on the activity of tumor-specific lymphocytes that are able to recognize and eliminate tumor cells. To get inside the tumor, lymphocytes first need to extravasate through tumor blood vessels. By facilitating lymphocyte trafficking to the tumor, TA-HEVs could play a key role in cancer immunity and immunotherapy.

The first descriptions of MECA-79^+^ HEV-like blood vessels in a human cancer setting were reported in cutaneous and gastric MALT lymphomas [51, 244, 245]. Given the known role of lymphocytes in the regulation of HEV-like blood vessels, it is not surprising that such vessels are present in extra-lymphoid tissues where malignant lymphoid cells accumulate. However, we now know that the development of MECA-79^+^ HEV-like blood vessels go far beyond lymphoid neoplasms, and they are in fact observed in many distinct human solid tumors [28], demonstrating that acquisition of HEV-specific attributes by tumor blood vessels is a widely conserved process in malignant tissues. Our initial observations showing the strong correlation between the density of MECA-79^+^ TA-HEVs and densities of tumor-infiltrating CD3^+^ T cells, CD8^+^ T cells, and CD20^+^ B cells in primary breast cancer and melanoma [28, 29], have been confirmed in many studies and extended to multiple human malignancies (Table 3). The density of MECA-79^+^ TA-HEVs is positively correlated with clinical parameters indicative of reduced tumor progression and invasion in primary melanoma [29, 246, 247] and with increased metastasis-free survival and overall survival in primary breast cancer [28]. MECA-79^+^ TA-HEVs are also associated with increased lymphocyte infiltration, progression free-survival and overall survival in head and neck cancer [248–250]. Moreover, combined high densities of MECA-79^+^ TA-HEVs and CD8^+^ T cells are a prognostic factor for overall survival in gastric cancer [251]. Together, these results suggest that TA-HEVs function as major gateways for lymphocyte infiltration into human tumors, thus promoting antitumor immune response and improving clinical outcome.

**Table 3:** MECA-79^+^ TA-HEVs in human cancer

Cancer type	Associated features	Clinical impact
*Primary tumors*		
Breast cancer [28, 252–255]	Progressive loss during ductal carcinoma progression from in situ to invasive; Associated with DC-LAMP^+^ DCs, T cell and B cell infiltrations; Associated with T_N_ and T_CM_ infiltration; Detected in > 74% of tumors (*n* = 127); Presence of TLSs in some tumors	Associated with increased DFS, MFS and OS; associated with pCR in triple-negative breast cancer patients treated with neoadjuvant chemotherapy
Melanoma [28, 29, 246, 247, 256, 257]	Progressive loss during tumor progression; Associated with DC-LAMP^+^ DCs, T cell and B cell infiltrations; Detected in > 66% of tumors (*n* = 225); Present in TLSs and outside TLSs in lymphocyte-rich areas	Correlate with tumor regression; No prognostic value for 5-year survival
Colorectal cancer [28, 258–261]	Associated with MSI^high^ colon cancer; Associated with T cell infiltration; Presence of TLSs	No prognostic value for 5-year survival
Lung cancer [28, 262–266]	Colocalized with CD62L^+^ lymphocytes; Present in TLSs and outside TLSs in lymphocyte-rich areas	
Testicular seminoma [267]	Preferentially associated with T cells; Co-expression of ICAM-1 but not VCAM-1 and MAdCAM-1; Co-expression of sLex epitope HECA-452; Associated with binding of E-selectin-IgM chimera	
Papillary thyroid carcinoma [268]	Preferentially associated with T cells, particularly CD8^+^ T cells; Co-expression of ICAM-1 but not VCAM-1 and MAdCAM-1; Co-expression of sLex epitope HECA-452; Associated with binding of E-selectin-IgM chimera	
Prostate [252, 269]	Present at different stages of cancer and in samples from patients with spontaneous remission; Presence of TLSs	
Urothelial bladder cancer [270]	Presence of TLSs in high-grade tumors	
Gastric cancer [251, 252, 271, 272]	Presence of TLSs	Combined high CD8^+^ T cell and MECA-79^+^ HEV-like blood vessel densities are associated with increased OS
Pancreatic cancer [252, 273–275]	Presence of TLSs	
Clear cell renal cell carcinoma [276]	Presence of TLSs	
Soft-tissue sarcomas [277]	Presence of TLSs	
Ovarian cancer [28, 252, 278]	Presence of TLSs	
Head and neck cancer [138, 248–250, 279]	Progressive loss during progression from T1 to T4 stages; Associated with T cell and B cell infiltrations; Presence of TLSs	Associated with increased DSS Associated with increased PFS and OS
Hepatocellular carcinoma [290]	Presence of TLSs	
*Metastases*		
Skin metastasis (melanoma) [246, 256]	Presence of TLSs	
Lung metastasis (colorectal cancer) [280]	Presence of TLSs	
Lung metastasis (renal cell carcinoma) [280]	Presence of TLSs	
Lung metastasis (breast cancer) [252]		
*Lymphomas*		
Cutaneous lymphomas [51, 245]		
Primary prostatic lymphomas [281]		
Gastric B-cell lymphomas of mucosa-associated lymphoid tissue [236, 244]	Co-expression of MAdCAM-1 and sLex epitope HECA-452	

*DC-LAMP* dendritic cell lysosomal associated membrane glycoprotein, *DFS* disease-free survival, *DSS* disease-specific survival, *HECA-452* mAb recognizing non-sulfated sLex, *MFS* metastasis-free survival, *OS* overall survival, *pCR* pathological complete response, *PFS* progression-free survival, *T*_*CM*_ central memory T cells, *T*_*N*_ naive T cells, *TLSs* B cell-rich tertiary lymphoid structures

**Fig. 5:**
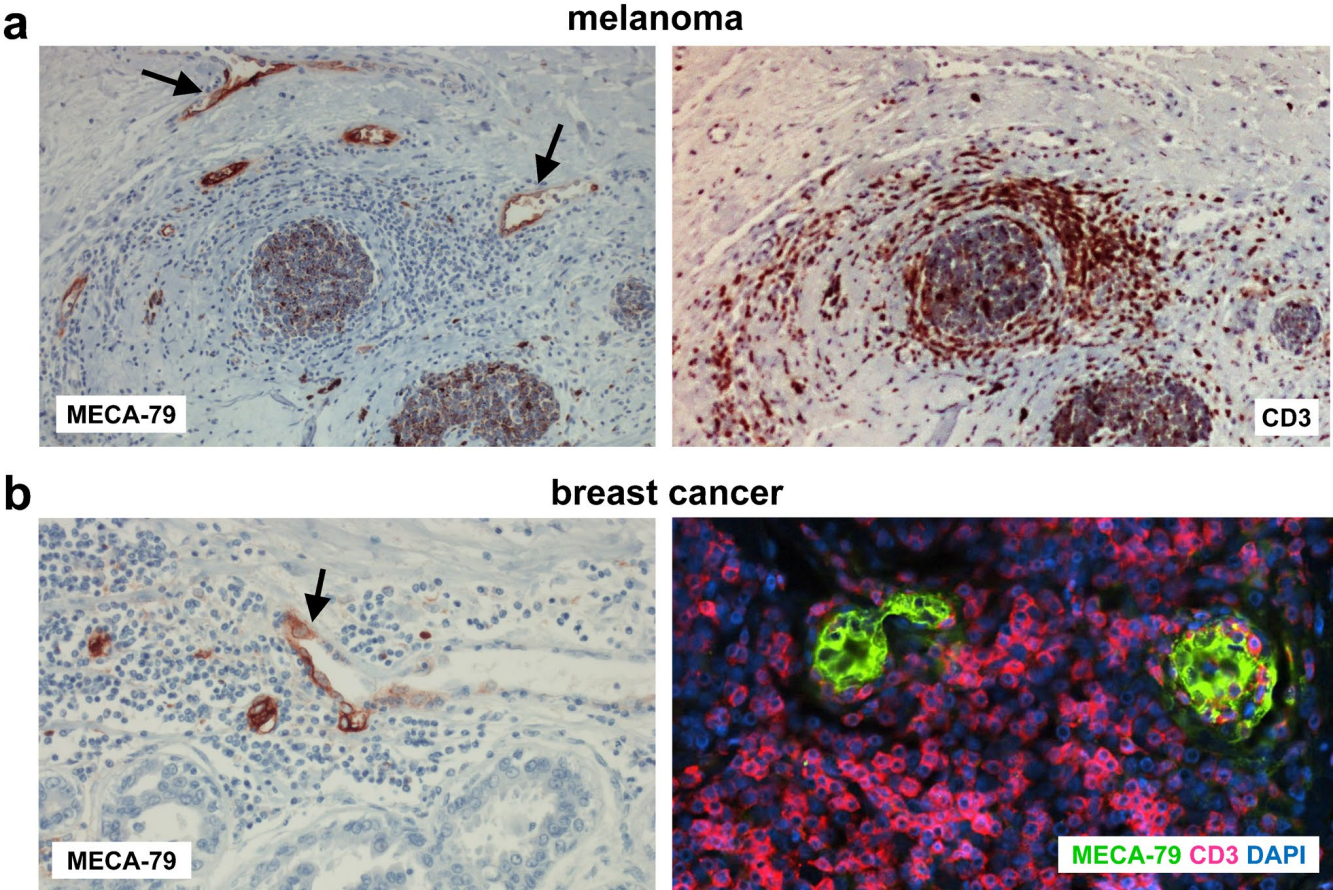
TA-HEVs and T cell infiltration in human primary melanoma and breast cancer. **a** MECA-79^+^ TA-HEVs in human primary melanoma. TA-HEVs are present in a regressing tumor area infiltrated by CD3^+^ T cells. **b** MECA-79^+^ TA-HEVs in human primary breast cancer. TA-HEVs are present in tumor areas infiltrated by CD3^+^ T cells. MECA-79 staining is more intense on the side of the blood vessels in contact with the lymphocytic infiltrate (arrows). See original references from Martinet, Garrido et al. [28, 29]

MECA-79^+^ TA-HEVs express pan-endothelial cell markers such as CD31 or von Willebrand factor (vWF) but also the marker DARC, indicating that they likely derive from post-capillary venules similar to HEV-like blood vessels in chronic inflammation [28, 29]. Endothelial cells lining human MECA-79^+^ TA-HEVs exhibit a cuboidal appearance reminiscent of the plump morphology of LN HECs (Fig. 5), but they can also display a flat morphology, frequently associated with a dilated vessel lumen [138, 246, 249]. As observed in chronic inflammatory diseases, MECA-79^+^ TA-HEVs might encompass a wide spectrum of HEV-like blood vessels with different degrees of maturation. Besides human studies, a main part of our knowledge on TA-HEVs come from studies performed in mouse models. Table 4 lists the different mouse tumors exhibiting MECA-79^+^ TA-HEVs. MECA-79^+^ TA-HEVs were first observed in mice following various treatments such as adoptive transfer of CD8^+^ T cells, administration of tumor-targeted LTα or genetic depletion of Foxp3^+^ regulatory T cells (Tregs) [282–285]. However, Engelhard et al. demonstrated that, in some tumor models, MECA-79^+^ TA-HEVs can spontaneously develop in the tumor microenvironment in the absence of any treatment [286]. Importantly, TA-HEVs are induced in different types of tumors, including subcutaneous transplanted tumors, orthotopically transplanted tumors and genetically engineered tumor models [252, 266, 286, 287]. In contrast to their human counterparts, endothelial cells lining mouse MECA-79^+^ TA-HEVs generally do not exhibit the HEV-specific cuboidal shape and they are characterized by a flat morphology associated with reduced expression of MECA-79 in comparison with LN HEVs [282, 286]. Nevertheless, they are surrounded by high numbers of lymphocytes akin to human TA-HEVs, providing an early clue to their functional significance [286]. Based on short-term in vivo homing assays, it was shown that MECA-79^+^ TA-HEVs are associated with the recruitment of naive lymphocytes into tumors [286], suggesting that LN HEV functional properties are conserved to some degree by TA-HEVs, and that HEV-mediated homing of lymphocytes in LN might be recapitulated in tumors. This later possibility is also supported by human studies in which high densities of MECA-79^+^ TA-HEVs correlated with increased infiltration of naive and central memory T cells [28].

**Table 4:** MECA-79^+^ TA-HEVs in mouse tumor tissues

Tumor type	Condition	Associated features
*Spontaneous induction*		
B16F1 subcutaneous tumors [286]		
Orthotopic 4T1 mammary carcinoma [252]		
Orthotopic LLC lung carcinoma [266]		
Orthotopic PyMT mammary carcinoma [287]		Increased with anti-VEGFR-2 + anti-PD-L1 or anti-VEGFR-2 + anti-PD-L1 + agonistic anti-LTβR treatment; LTβR-Ig treatment abolishes increase of MECA-79^+^ TA-HEVs after anti-VEGFR-2 + anti-PD-L1 treatment
Colorectal cancer [259]	AOM/DSS induction	Presence of TLSs
Orthotopic panc02 pancreatic tumors [252]		
*Genetically modified mice and cell lines*		
Melanoma [286]	BRAF^V600E^PTEN^−/−^ mice	
Melanoma [288]	Myct1^−/−^ mice	
Lung adenocarcinoma [289]	KP mice (Kras^G12D^, Trp53^−/−^)	Associated with TLSs and local proliferation of CD8^+^ T cells after depletion of Tregs
Hepatocellular carcinoma [290]	IKKβ(EE)^Hep^ mice	Present in TLSs containing malignant hepatocyte progenitor cells
MCA-induced fibrosarcomas [282, 291]	Depletion of FoxP3^+^ Tregs in Foxp3^DTR^ mice	Present in CD11c.DOG mice and after LTβR-Ig treatment; Absent after CD8^+^ T cell depletion and reduced after TNFR2-Ig, anti-TNFα or anti-LTα treatment
LLC-OVA subcutaneous and intraperitoneal tumors [286]	Ovalbumin-expressing lung carcinoma cells	
B16F1-AAD subcutaneous and intraperitoneal tumors [286]	Tyrosinase-expressing melanoma cells	
B16F1-OVA subcutaneous and intraperitoneal tumors [286]	Ovalbumin-expressing melanoma cells	Absent in Rag1 and Rag2^−/−^ mice, and phenotypic rescue with adoptive transfer of WT, IFNγ^−/−^ and TNFα^−/−^ CD8^+^ T cells but not LTα^−/−^ CD8^+^ T cells; Absent in TNFR1/2^−/−^ mice; Present in IFNγ^−/−^ and TNFα^−/−^ mice, and after LTβR-Ig treatment; Presence of TLSs in i.p. tumors but not in s.c. tumors
B16F10-CCL21 subcutaneous tumors [292]	CCL21-expressing melanoma cells	Associated with CCL21-induced immune tolerance
J558L-LTα subcutaneous tumors [293]	LTα-expressing plasmacytoma cells	Present in NUDE and SCID mice
*Therapeutic induction*		
B16-GD_2_ subcutaneous tumors [283, 284]	GD_2_-expressing melanoma cells; Ch14.18-LTα (GD_2_-targeted lymphotoxin α)	Present in LTα^−/−^ mice
Pancreatic tumors (RIP1-Tag5 mice) [285, 294]	LIGHT-CGKRK (tumor blood vessels-targeted LIGHT)	Presence of TLSs
	Adoptive transfer of ex vivo activated lymphocytes in irradiated recipients	
LLC subcutaneous tumors [294, 295]	LIGHT-CGKRK; PARP inhibitor (BMN673)	
B16F10 lung metastases [296]	LIGHT-CGKRK	Increased when LIGHT-CGKRK is combined with anti-PD-1 treatment; Presence of TLSs
Orthotopic NFpp10 glioblastoma [287, 297]	LIGHT-CGKRK; Anti-VEGFR-2 + anti-PD-L1 + agonistic anti-LTβR	Increased when LIGHT-CGKRK is combined with anti-VEGFR-2 + anti-PD-L1 treatment
Orthotopic KPC1199 pancreatic ductal adenocarcinoma [298]	Tumor-targeted liposome carrying plasmids encoding LIGHT	Presence of TLSs
Pancreatic neuroendocrine tumors (RT2-PNET mice) [287]	Anti-VEGFR-2 + anti-PD-L1	
B16F10 subcutaneous tumors [299]	STING agonist (ADU-S100)	Absent in STING^−/−^ mice
MCA205 subcutaneous tumors, MC38 subcutaneous tumors [300]	Intratumoral injection of T-bet-expressing dendritic cells	

*AOM* azoxymethane, *DSS* dextran sodium sulphate, *MCA* 3-methylcholanthrene, *DTR* diphtheria toxin receptor, *LT* lymphotoxin α, *LTαβ* lymphotoxin α and β, *GD*_*2*_ disialoganglioside, *Tregs* regulatory T cells, *RIP* rat insulin promoter, *RT2* RIP1-Tag2, *TLSs* B cell-rich tertiary lymphoid structures, *VEGFR-2* vascular endothelial growth factor receptor 2

**Fig. 6:**
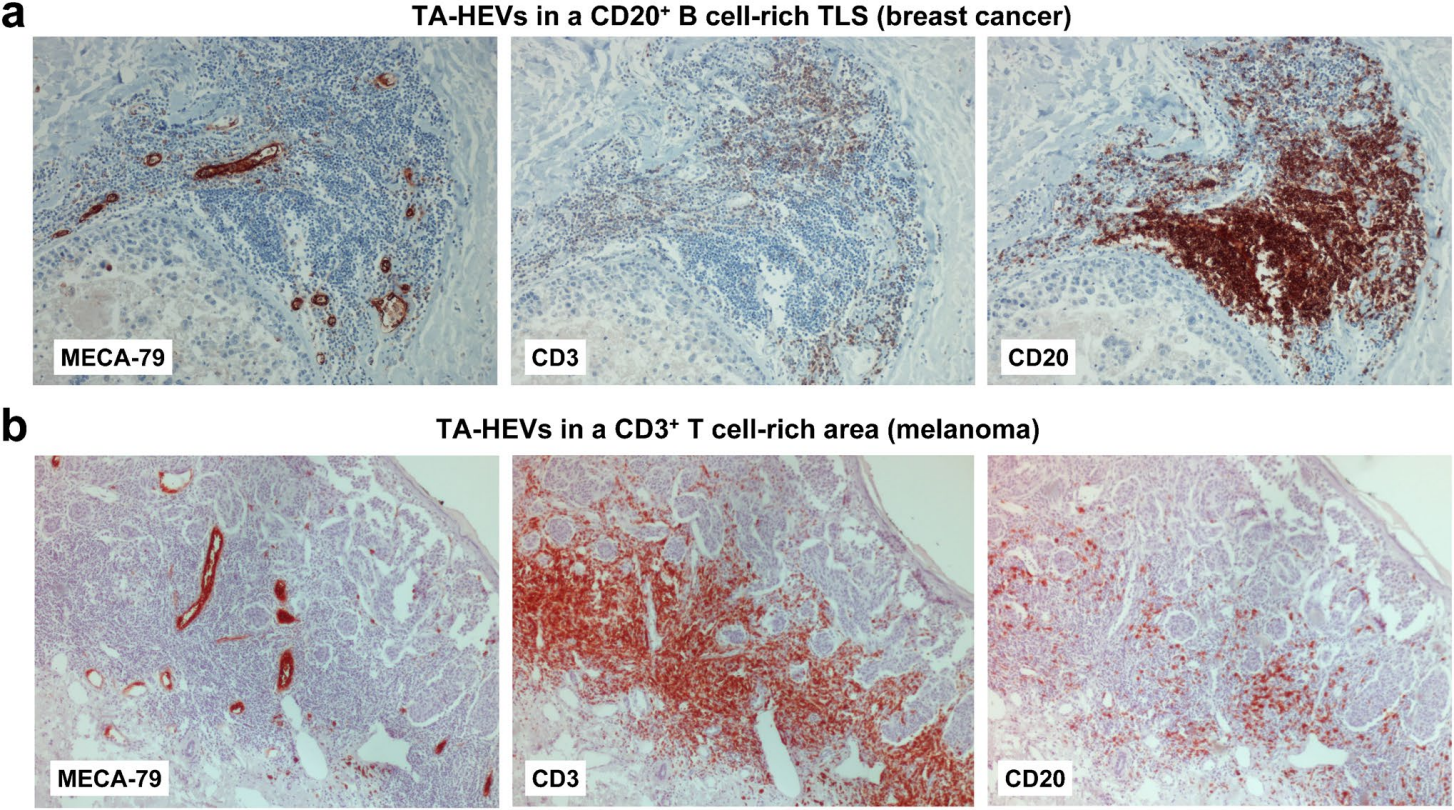
TA-HEVs are present in both T cell-rich areas and B cell-rich TLSs. **a** MECA-79^+^ TA-HEVs in human primary breast cancer. TA-HEVs are present in a tumor area highly infiltrated by CD20^+^ B cells. These lymphoid aggregates enriched in B cells are designated B cell-rich TLSs. **b** MECA-79^+^ TA-HEVs in human primary melanoma. TA-HEVs are present in a tumor area highly infiltrated by CD3^+^ T cells and by some CD20^+^ B cells with no apparent organization into TLSs. See original references from Martinet, Garrido et al. [28, 29]

The recruitment of naive T cells into tumors is of particular interest since their priming and subsequent conversion into effectors can be realized directly within the tumor [301, 302]. Bypassing activation of naive T cells in LNs is proposed to accelerate and foster antitumor response and is likely to occur in tumor TLSs [303, 304]. TA-HEVs are present in B-cell rich TLSs that develop in some human tumors (Fig. 6a) [277, 303, 305] and might contribute to their function in a similar way to HEV-like blood vessels in TLSs from chronic inflammatory diseases [306–310]. However, TA-HEVs and tumor-associated TLSs are two distinct elements. Indeed, TA-HEVs are more frequent than TLSs in human tumors. For instance, we detected MECA-79^+^ TA-HEVs in > 74% of primary breast tumors (*n* = 127) [28], whereas TLSs were found in only 37% of tumors in a cohort of 248 breast cancer patients [311]. In primary melanoma, we found TA-HEVs in > 66% of tumors (*n* = 225) [29], whereas TLSs are rarely detected in primary melanoma lesions [256]. Indeed, MECA-79^+^ TA-HEVs are often found in T-cell rich areas containing DCs but no B cell follicles (Fig. 6b) [29, 247, 253, 256, 262]. These structures enriched in T cells and DCs, that are highly similar to the T-cell zones of lymphoid tissues, may provide a supportive niche for CD8^+^ T cells in human tumors [312]. Jansen et al. also reported the presence of TLSs in their tumor samples [312], but these TLSs were located in distinct areas and were mainly composed of B cells, suggesting that CD8^+^ T cell- and B cell-dependent responses may occur in distinct structures in human tumors. Whether TA-HEVs are an integral part of the T-cell oriented structures remains to be confirmed, but the known association of TA-HEVs with both T cells and DCs in human tumors is in agreement with this hypothesis [28, 29, 253].

Importantly, although TLSs containing MECA-79^+^ TA-HEVs are associated with a favorable clinical outcome in most cancer types [303], they may be detrimental in some instances. Hepatocellular carcinoma (HCC) is an inflammation-driven cancer characterized by abundant TLSs that were associated with increased risk of recurrence [290]. In fact, Finkin et al. demonstrated that TLSs could serve as niches for malignant hepatocyte progenitors in an HCC mouse model, suggesting that TLSs could support tumor progression in inflammation-dependent tumors. In addition, TLSs have been reported to be privileged sites for Treg accumulation in some mouse tumor models [289, 292]. Therefore, TLSs are generally associated with antitumor functions but could also shelter cells promoting tumor growth, showing that the impact of TLSs on prognosis is dependent on cancer types.

### Mechanisms regulating the development of TA-HEVs in cancer

Induction of TA-HEVs in solid tumors highlights the remarkable capacity of immune cells to modify their target tissue to maximize the immune response, even in highly hostile microenvironments. Despite similarities with HEV-like blood vessels of chronic inflammatory diseases, the mechanisms regulating TA-HEV development also have distinctive features related to the particular nature of solid tumors. Determining the cellular actors and molecular signals triggering the HEV differentiation program in tumor blood vessels is essential to better define their role in antitumor immunity, and to provide important insights for the design of novel therapeutic approaches based on TA-HEV induction.

Because DCs control HEV phenotype and function in lymph nodes [94] and since they are frequently associated with TA-HEVs in human melanoma and breast cancer [29, 253], lymphotoxin-expressing DCs were initially proposed as critical regulators of TA-HEVs in humans [313]. However, results obtained in mouse models point towards a more dominant role for lymphocytes. As observed for HEV-like blood vessels in models of chronic inflammation, MECA-79^+^ TA-HEVs are lost in tumors grown in lymphocyte-deficient *Rag2*^*−/−*^ mice [286]. Moreover, adoptive transfer of CD8^+^ T cells is sufficient to induce development of MECA-79^+^ TA-HEVs in *Rag2*^*−/−*^ mice, indicating that CD8^+^ T cells could be major inducers of TA-HEVs in tumors. In contrast, Tregs, which are known as major immunosuppressive cells in the tumor microenvironment, seem to limit TA-HEV development in tumors as revealed by the induction of MECA-79^+^ TA-HEVs following depletion of Foxp3-expressing cells in Foxp3^DTR^ mice [282, 291]. Whether Tregs inhibit HEV neogenesis via direct action on tumor blood vessels or indirectly via inhibition of lymphocyte subsets critical for TA-HEV development is currently unknown. Interestingly, depletion of CD8^+^ T cells was shown to abrogate MECA-79^+^ TA-HEV induction consecutive to Treg depletion [291], confirming the important role of CD8^+^ T cells in the regulation of TA-HEVs. The increased MECA-79 staining of TA-HEV endothelial cells in close proximity with CD3^+^ T cells in human primary melanoma (Fig. 5) further highlights the importance of lymphocytes in TA-HEV regulation. Together, these results indicate that lymphocytes are able to induce specialized blood vessels facilitating their trafficking into tumors, revealing an important immune-vascular crosstalk in favor of antitumor immunity.

MECA-79^+^ TA-HEVs observed in mouse tumor models express low amounts of MECA-79 antigens at their surface. In line with this immature phenotype, their development is critically dependent on TNFR1/2 signaling and not LTβR pathway. Indeed, TNFR1/2^−/−^ mice do not develop TA-HEVs while treatment with LTβR-Ig has no impact on the development of MECA-79^+^ TA-HEVs in wild type mice [286]. Similar findings were obtained for TA-HEVs induced following Treg depletion as their development is blocked by TNFR-Ig fusion protein but not LTβR-Ig [291]. Because TA-HEVs are not affected in TNFα^−/−^ mice and because LTα^−/−^ CD8^+^ T cells induce significantly less MECA-79^+^ TA-HEVs in lymphocyte-deficient mice than wild type CD8^+^ T cells, LTα, a TNFR1 ligand, was proposed as a key mediator for the development of TA-HEVs [286]. Consistent with this, tumors of mice treated with tumor-targeted LTα or bearing cancer cells genetically engineered to secrete LTα develop MECA-79^+^ TA-HEVs whereas control tumors are devoid of such vessels [283, 284, 293]. Although LTβR signaling is not required for the development of most TA-HEVs observed in mouse tumors, several reports indicate that stimulation of this receptor leads to the development of MECA-79^+^ TA-HEVs. Indeed, treatment with the LTβR ligand LIGHT or LTβR agonistic antibodies is sufficient to induce TA-HEVs in mouse tumors [287, 294, 296, 297]. Therefore, LTβR signaling is dispensable for the development of mouse TA-HEVs, which probably explains their relative immaturity in comparison to LN HEVs, but therapeutic targeting of this receptor induces TA-HEVs. The two-step differentiation model of HEV-like blood vessels in chronic inflammation could also be true for TA-HEVs (Fig. 4). However, additional studies are required to determine if LTβR stimulation is actually able to increase the degree of maturation of mouse TA-HEVs. Signaling through LTβR may be critical for induction of fully mature TA-HEVs in tumors.

The association of histological examinations of TA-HEVs with clinical parameters indicate that TA-HEV density is dependent on the tumor stage in humans. Breslow tumor thickness is used as a prognostic biomarker for staging primary cutaneous melanomas and it was shown that densities of MECA-79^+^ TA-HEVs are inversely correlated with Breslow thickness, indicating that TA-HEVs are more abundant during the initial stages of melanoma [29]. Analysis of head and neck cancer with the tumor-node-metastasis (TNM) staging system revealed that T1 tumors exhibit higher densities of TA-HEVs as compared to tumors of later stages [248, 249]. Interestingly, the progression from in situ to invasive ductal carcinoma is associated with a progressive loss of TA-HEVs in breast cancer [253]. These correlations suggest that induction of TA-HEVs is maximal during the initial stages of tumor development when the immune response is likely to be the highest. In fact, results obtained in transgenic mice expressing the oncoprotein Tag (simian virus 40 large T antigen) under control of the rat insulin gene regulatory region (RIP1-Tag5 mice) corroborate the observations obtained in human tumors. In RIP1-Tag5 mice, Tag expression in the insulin-producing cells of the pancreatic islets induces multistage carcinogenesis of pancreatic islets starting with benign hyperplasia and ending with the development of solid tumors and premature death [314]. In striking contrast to the highly infiltrated hyperplastic islets that contain MECA-79^+^ TA-HEVs, tumors are poorly infiltrated by lymphocytes and do not develop TA-HEVs although they are highly vascularized [212]. These observations confirmed that tumor progression influences the presence of TA-HEVs and suggested that tumor immunogenicity may control induction and/or maintenance of TA-HEVs. In agreement with this later possibility, mice with B16F1 tumors contain far less MECA-79^+^ TA-HEVs than mice with B16F1 tumors expressing ovalbumin that are known to elicit robust lymphocyte-dependent antitumor response because of the high level of antigenicity of ovalbumin [286]. Therefore, the presence of TA-HEVs in tumors might be a good proxy to evaluate the intensity of the ongoing antitumor immune response. The loss of TA-HEVs during tumor progression may be due to the loss of strong neoantigens by cancer immunoediting [315, 316]. However, the impact of cancer immunoediting on TA-HEVs is currently unknown.

### Therapeutic induction of TA-HEVs in cancer

**Fig. 7:**
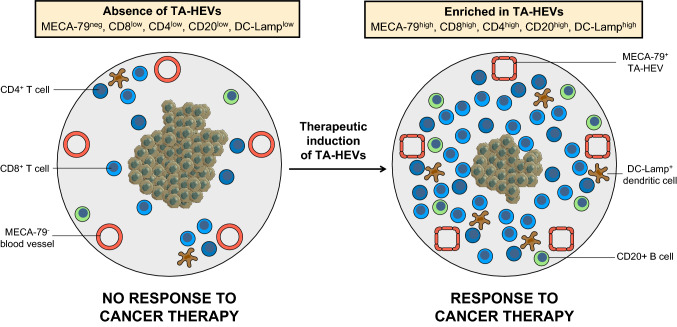
Therapeutic induction of TA-HEVs for cancer therapy. Induction of MECA-79^+^ TA-HEVs in the tumor microenvironment may increase infiltration of various subsets of CD8^+^ and CD4^+^ T cells, as well as CD20^+^ B cells, and may improve antitumor immunity and efficacy of various cancer treatments, including immunotherapies with immune checkpoint inhibitors, adoptive T cell therapy or vaccines, but also potentially targeted therapies and conventional cancer therapies (radiotherapy, chemotherapy)

Trafficking of lymphocytes to tumors is critical for antitumor immunity and cancer immunotherapy with immune checkpoint inhibitors (ICIs), vaccines or adoptive T cell therapy (ACT) [317–321]. Tumor-infiltrating lymphocytes (TILs) are associated with improved clinical outcome in many cancers, and the presence of high numbers of CD8^+^ T cells in human tumors is predictive of therapeutic response to cancer treatments, especially to ICIs [322–325]. However, the mechanisms governing the magnitude of the CD8^+^ T cell response remain incompletely defined. Why some tumors have high CD8^+^ T cell infiltration while others have poor infiltration is not entirely clear. Increasing the density and maturation of MECA-79^+^ TA-HEVs in the tumor microenvironment may enhance lymphocyte trafficking to tumors and improve the efficacy of various cancer treatments (Fig. 7), including immunotherapies with ICIs, ACT or vaccines, but also potentially targeted therapies and conventional cancer therapies (radiotherapy, chemotherapy).

Checkpoint blockade therapy with anti-PD-1 and anti-CTLA-4 antibodies provides remarkable and durable responses for many patients across different types of cancer [317, 318, 320]. However, ICIs do not benefit all patients and novel therapeutic strategies are required for increasing their efficacy. Recent studies indicate that a subset of tumor-reactive CD8^+^ T cells may be critical for antitumor immunity at baseline and also for response to cancer immunotherapies with ICIs [312, 326–328]. This particular T cell subset encompasses less differentiated and less dysfunctional (exhausted) CD8^+^ T cells designated stem-like CD8^+^ T cells because of their capacity to self-renew while being able to generate more differentiated effector CD8^+^ T cells. Interestingly, analyses of T cells in patients treated with ICIs revealed that continuous recruitment of fresh and less exhausted T cells from the periphery into the tumor may be important for clinical response [329, 330]. Together, these findings suggest that strategies aiming to ameliorate the migration of peripheral stem-like CD8^+^ T cells into tumors could result in increased numbers of patients responding to ICIs. As specialized blood vessels for lymphocyte trafficking, TA-HEVs may be major gateways for entry of stem-like CD8^+^ T cells into tumors, and their therapeutic modulation could enhance the infiltration of these critical cells, thus increasing the efficacy of ICIs. Infiltration of naïve and central memory CD8^+^ T cells, CD4^+^ T cells and B cells through TA-HEVs may also play important roles in the response to ICIs [277, 305] and other forms of cancer therapies (Fig. 7).

As expected, LTβR agonists are potent inducers of TA-HEVs in tumors. Targeting LIGHT directly to tumor blood vessels via fusion to vascular targeting peptides (VTP) induces MECA-79^+^ TA-HEVs in various mouse tumor models [294, 296, 297]. Similar results were obtained with a tumor-targeted nanoparticle co-loaded with an anti-fibrotic molecule and a plasmid encoding LIGHT [298]. LIGHT-induced development of TA-HEVs is associated with increased lymphocyte infiltration and response to ICIs [294]. Notably, LIGHT-associated therapies were also shown to overcome resistance to anti-PD-1 or anti-PD-L1 monotherapies and to sensitize refractory lung metastases to anti-PD-1 immunotherapy [296, 298, 331]. LTβR agonistic antibodies (anti-LTβR) were also reported to induce MECA-79^+^ TA-HEV and to enhance lymphocyte infiltration in distinct mouse tumor models, and treatment with anti-LTβR enabled response to anti-VEGFR2 and anti-PD-L1 combination therapy in a recalcitrant glioblastoma model [287]. Different cell types express LTβR in the tumor microenvironment. Therapeutic stimulation of LTβR with LIGHT or LTβR agonistic antibodies may thus reprogram intratumoral stromal cells and dendritic cells, in addition to blood vessel endothelial cells [332]. Along with LTβR stimulation, TNFR1 stimulation may also provide an effective way to induce TA-HEVs. Indeed, previous studies with a tumor-targeted antibody-LTα fusion protein showed that stimulating TNFR1 in the tumor microenvironment was able to induce MECA-79^+^ TA-HEV and to eradicate established tumors [283, 284]. Whether these MECA-79^+^ tumor blood vessels are identical to MECA-79^+^ TA-HEVs induced through LTβR stimulation warrants further studies, but in both instances, neogenesis of TA-HEVs correlated with a robust lymphocyte-mediated antitumor response. Intriguingly, other agents targeting signaling pathways not related to HEV biology induce TA-HEVs. For instance, intratumoral injection of STING agonists (ADU-S100) or treatment with a PARP inhibitor (BMN 673) both induce MECA-79^+^ TA-HEVs in mouse tumor models [295, 299]. Together these studies in mice provide a proof-of-concept that induction of TA-HEVs within tumors can unleash lymphocyte-dependent immunity and improve therapeutic outcomes.

Therapeutic induction of TA-HEVs in tumors might enhance trafficking of endogenous lymphocytes but also of adoptively transferred lymphocytes. If cell-based immunotherapies with chimeric antigen receptor (CAR) T cells are showing great promises in the treatment of hematological malignancies (e.g. CD19-targeted CAR T cells for B-cell acute lymphocytic leukemia), they are usually ineffective for treatment of solid tumors [333]. Pre-conditioning the tumor vasculature for maximal lymphocyte trafficking through induction of TA-HEVs could thus provide therapeutic benefits in ACT immunotherapy of solid tumors, including with CAR T cells. Interestingly, the success of ACT using ex vivo-expanded autologous TILs is dependent on the presence of stem-like CD8^+^ T cells within transferred cells, demonstrating the crucial role of these particular CD8^+^ T cells in cell-based immunotherapies in human cancer [334]. In addition, several studies demonstrated that transferring less-differentiated CD8^+^ T cells (e.g. central memory T cells) elicit better antitumor responses during therapeutic ACT in mouse tumor models [335–337]. Therefore, the unique ability of TA-HEVs to capture naive and naive-like lymphocytes might be particularly valuable for ACT immunotherapy, especially when using early differentiated cells that express L-selectin and CCR7.

## Conclusion

Blood vessels that are structurally and phenotypically similar to HEVs from lymphoid organs appear in non-lymphoid tissues during chronic inflammation and cancer. HEV-like blood vessels in chronically inflamed tissues and TA-HEVs in tumors are associated with lymphocyte infiltration similar to lymphoid tissue HEVs, indicating that induction of specialized blood vessels for lymphocyte trafficking is a universal property of tissues exposed to intense lymphocyte activity. In chronic inflammatory diseases, HEV-like blood vessels facilitate influx of pathological lymphocytes, leading to amplification and maintenance of chronic inflammation. In contrast, TA-HEVs are generally beneficial in cancer, showing that the clinical significance of ectopic HEV-like blood vessels is highly dependent on the pathological context.

In the past 30 years, there has been considerable progress in our understanding of the mechanisms regulating the phenotype and function of HEVs in LNs, both at steady state and following immune challenge. However, several questions remain regarding the phenotype and functionality of HEV-like blood vessels and TA-HEVs. For instance, the use of intravital microscopy, which is the only experimental approach enabling visualization of lymphocyte recruitment through blood vessels in vivo [52], will be crucial to demonstrate the functional competence of these vessels. In particular, determining the relative contribution of MECA-79^+^ blood vessels versus MECA-79^−^ blood vessels will be important to confirm the increased capacity of HEV-like blood vessels and TA-HEVs to mediate lymphocyte recruitment into tissues. Recent transcriptomic analyses of mouse MECA-79^+^ HECs delineate the HEV phenotype in homeostatic and inflamed LNs [40, 53, 54]. Investigating the transcriptomes of endothelial cells lining HEV-like blood vessels and TA-HEVs and comparing them with those of LN HECs and non-HEV endothelial cells in mouse and human tissues could provide great insights about potential pathways for modulation of these vessels in chronic inflammation and cancer.

Great promises stem from the potential of TA-HEVs to increase lymphocyte trafficking into tumors, especially for cancer immunotherapy, which has to face unmet clinical needs. Because of their unique ability to mediate the recruitment of L-selectin-expressing lymphocytes, therapeutic induction of MECA-79^+^ TA-HEVs could not only increase lymphocyte trafficking quantitatively, but also qualitatively by enabling the entry of specific lymphocyte subsets that may be critical for antitumor immunity. These may include naive, central memory and stem-like CD8^+^ T cells, but also CD4^+^ T cells and B cells [277, 305, 338]. However, we have to learn lessons from the clinical failure of therapeutic agents targeting HEV-like blood vessels in chronic inflammatory diseases. Solely inducing TA-HEVs will be probably insufficient to obtain significant clinical responses, but therapeutic combinations with ICIs, ACT or other forms of cancer therapy are likely to provide important therapeutic benefits for cancer patients.

In chronic inflammation, MECA-79^+^ HEV-like blood vessels accurately accompany lymphocyte-dependent activity and disease progression. Similar findings in solid tumors mean that MECA-79^+^ TA-HEVs go along with antitumor immune response and may represent a biomarker to identify highly immunogenic tumors that are more likely to respond to cancer immunotherapies. Indeed, it is important to identify biomarkers predicting response to ICIs because they are widely used for metastatic patients who are frequently non-responsive and develop severe immune-related adverse events [318, 339]. Since MECA-79^+^ TA-HEVs are present in metastatic lesions, there is an urgent need to investigate their capacity to predict response to ICIs in cancer patients.

In this article, we presented a comprehensive review on HEVs and HEV-like blood vessels in immunity, inflammation and cancer. HEVs in lymphoid organs have fascinated many researchers over the past century. We are convinced that HEVs and HEV-like blood vessels will continue to attract the interest of scientists and clinicians in the next decades, particularly those working in the areas of vascular biology (angiogenesis), immunology, inflammation, cancer biology (tumor microenvironment) and cancer immunotherapy. Although many aspects of HEV-like blood vessels are still to be discovered, their therapeutic modulation already offers promising avenues, especially for cancer treatment.
